# Fine metagenomic profile of the Mediterranean stratified and mixed water columns revealed by assembly and recruitment

**DOI:** 10.1186/s40168-018-0513-5

**Published:** 2018-07-10

**Authors:** Jose M. Haro-Moreno, Mario López-Pérez, José R. de la Torre, Antonio Picazo, Antonio Camacho, Francisco Rodriguez-Valera

**Affiliations:** 1 Evolutionary Genomics Group, División de Microbiología, Universidad Miguel Hernández, Apartado 18, San Juan de Alicante, 03550 Alicante, Spain; 2 Department of Biology, San Francisco State University, San Francisco, CA 94132 USA; 3 Cavanilles Institute of Biodiversity and Evolutionary Biology, University of Valencia, Burjassot, E-46100 Valencia, Spain

**Keywords:** Photic zone, Deep chlorophyll maximum, Mediterranean, Stratification, Stenobathic

## Abstract

**Background:**

The photic zone of aquatic habitats is subjected to strong physicochemical gradients. To analyze the fine-scale variations in the marine microbiome, we collected seven samples from a single offshore location in the Mediterranean at 15 m depth intervals during a period of strong stratification, as well as two more samples during the winter when the photic water column was mixed. We were able to recover 94 new metagenome-assembled genomes (MAGs) from these metagenomes and examine the distribution of key marine microbes within the photic zone using metagenomic recruitment.

**Results:**

Our results showed significant differences in the microbial composition of different layers within the stratified photic water column. The majority of microorganisms were confined to discreet horizontal layers of no more than 30 m (stenobathic). Only a few such as members of the SAR11 clade appeared at all depths (eurybathic). During the winter mixing period, only some groups of bloomers such as *Pseudomonas* were favored. Although most microbes appeared in both seasons, some groups like the SAR116 clade and some Bacteroidetes and Verrucomicrobia seemed to disappear during the mixing period. Furthermore, we found that some microbes previously considered seasonal (e.g., Archaea or Actinobacteria) were living in deeper layers within the photic zone during the stratification period. A strong depth-related specialization was detected, not only at the taxonomic level but also at the functional level, even within the different clades, for the manipulation and uptake of specific polysaccharides. Rhodopsin sequences (green or blue) also showed narrow depth distributions that correlated with the taxonomy of the microbe in which they were found but not with depth.

**Conclusions:**

Although limited to a single location in the Mediterranean, this study has profound implications for our understanding of how marine microbial communities vary with depth within the photic zone when stratified. Our results highlight the importance of collecting samples at different depths in the water column when comparing seasonal variations and have important ramifications for global marine studies that most often take samples from only one single depth. Furthermore, our perspective and approaches (metagenomic assembly and recruitment) are broadly applicable to other metagenomic studies.

**Electronic supplementary material:**

The online version of this article (10.1186/s40168-018-0513-5) contains supplementary material, which is available to authorized users.

## Background

Stratified systems are widespread on Earth, from microbial mats to meromictic lakes and the temperate ocean. A common factor of all stratified systems is strong vertical physicochemical gradients [1, 2]. The large scale of the oceanic environment makes the upper 100 m seem relatively small. Nevertheless, this layer of the water column is one of the most biologically productive microbial habitats in the biosphere [3]. The open ocean is far from homogenous, and environmental conditions are strongly affected by the depth in the water column [4, 5]. As the depth increases, temperature declines, salinity increases, and the availability of nutrient dwindles. Among these factors, light attenuation is of paramount importance. The main divide in aquatic environments tends to be between the photic zone, where light allows for photosynthesis, and the aphotic zone, which is beyond the compensation depth and where the available light (if any) is insufficient to drive photosynthesis. The availability of light is critical for primary productivity and hence is the main limiting factor for organic matter production throughout the water column [6]. The differences in the microbiome between the photic and aphotic zones are well known using a variety of approaches [4, 7–9]. Studies at global ocean scales such as those derived from the Sorcerer II Global Ocean Sampling [10] or the more recent Tara Oceans expedition [11] have provided essential information on the composition, dynamics, and spatial distribution of surface ocean microbial communities. However, much less attention has been devoted to the differences in the vertical distribution of microbial communities. This lack of attention is particularly true of the microbial assemblages within the photic zone, where samples from a single depth are often considered representative of the complete photic water column. However, most offshore oceanic waters are permanently or seasonally stratified, sometimes as deep as hundreds of meters, which creates strong gradients of environmental parameters.

In the Mediterranean, the water column is seasonally stratified, typically from March to November. A characteristic and extensively studied phenomenon associated with this stratification is the formation of the deep chlorophyll maximum (DCM) [1], a maximum in chlorophyll concentration that is associated with an increase in bioavailable pools of nitrogen (N) and phosphorus (P) diffusing from the mixed layer below the seasonal thermocline [12]. In tropical waters, the DCM is a permanent feature, whereas in the Mediterranean and other temperate waters, the DCM is a seasonal phenomenon [13] that often appears between 45 and 70 m deep [14], depending on the degree of light penetration (dictated by the season of the year and biological productivity).

During late autumn and winter, the temperature decrease near the surface leads to vertical mixing of the water column and promotes the upwelling of nutrients (mainly dissolved organic carbon (DOC), P and N) from the mesopelagic to the euphotic zone [15]. The availability of these nutrients results in phytoplankton blooms during spring [15]. When these blooms decay, a large amount of nutrients is released, and this ecological disturbance reshapes the composition of the prokaryotic community [16–19]. The Mediterranean Sea is characterized by a relatively high temperature (> 13 °C) throughout the entire water column. Although the mixing depth is variable depending on the year, it is normally located beyond 200 m [20].

Previous studies have used denaturing gradient gel electrophoresis (DGGE) [21], catalyzed reporter deposition-fluorescence in situ hybridization procedure (CARD-FISH), and clone libraries [22] to demonstrate the seasonal variability of the prokaryotic community in the northwestern Mediterranean observatory located in Blanes Bay. However, most of these studies were based at one single depth (surface). Furthermore, variations within the community were predicted at the level of a class or at most at the level of genera, ignoring the fact that within the same species, different ecotypes have different niche specialization, and therefore, they are found at different depths, such as *Prochlorococcus* high-light and low-light ecotypes [23]. Besides, many used PCR of 16S rRNA genes introducing unknown biases, and many others relied on FISH where mismatches on the probes can underestimate the abundance of the different prokaryotic groups. Metagenome shotgun sequencing, genome reconstruction, and metagenomics recruitment can give us a glimpse of the uncultured community inhabiting in this region, and changes in their concentration among different samples can be followed at a much finer level.

Here, we have analyzed two temporal sampling efforts, one with samples collected during the stratified period every 15 m throughout the photic zone (down to 90 m) and the other with samples collected during the winter when the water column was mixed (at two depths, 20 and 80 m). To assess the variations in the community structure, we used genome-resolved metagenomics [24] to measure the recruitment of reconstructed and reference genomes at the different depths and conditions (stratified or mixed), at high similarity thresholds. This allowed the discrimination of different ecotypes within the same species. We detected marked stratification of ecotypes that reflects species adaptation to live at defined depth range. Furthermore, we detected a stable component of the photic zone microbial community, which was present regardless of the season or physicochemical parameters. Other microbes were more sensitive and appeared only in a specific season. Our results highlight the importance of collecting and comparing samples from multiple depths to understand the dynamics between mixed and stratified waters.

## Results and discussion

Seawater samples from six depths in the photic zone were collected at 15 m intervals (15, 30, 45, 60, 75, and 90 m) on a single day (between 8 am and noon) during the stratification period (15 October 2015). For the comparison between the photic and aphotic regions, we also collected another sample from 1000 m (the next day starting at 8 am) from the same site. Another two samples were collected on 27 January 2015, during the winter mixing at 20 and 80 m. With the exception of the 1000 m sample, all the samples were collected using a hose directly connected to the filtration apparatus to minimize processing time and to avoid bottle effects (Additional file 1: Figure S1). The metadata and sequencing results are described in Table 1.

**Table 1:** Summary statistics of the sampling, sequencing, and assembly parameters

	MedWinter-JAN2015-20m	MedWinter-JAN2015-80m	Med-OCT2015-15m	Med-OCT2015-30m	Med-OCT2015-45m	Med-OCT2015-60m	Med-OCT2015-75m	Med-OCT2015-90m	Med-OCT2015-1000m
Sampling parameters									
Sampling data	1/27/2015		10/15/2015						10/16/2015
Collection depth (m)	20	80	15	30	45	60	75	90	1000
Sea bottom depth (m)	200		2647						
Size fraction (μm)	5–0.22		5–0.22						
Latitude (N)	38.06851		37.35361						
Longitude (W)	0.231994		0.286194						
Environmental parameters									
Temperature (°C)	14.50	14.40	22.90	18.40	15.80	14.50	14.00	13.80	13.10
Chlorophyll (mg/m^3^)	0.46	0.21	0.10	0.24	0.78	0.36	0.26	0.08	0.01
Oxygen (mg/L)	9.59	9.42	7.09	8.77	9.00	7.66	7.16	6.88	6.10
TOC (mgC/L)	1.23	1.03	2.43	2.17	1.46	1.43	1.36	1.35	0.84
PO_4_^3−^ (μM)	0.12	0.08	0.06	0.07	0.10	0.08	0.12	0.22	0.39
Total P (μM)	0.15	0.12	0.10	0.12	0.14	0.12	0.16	0.25	0.45
NO_x_ (μM)	3.26	3.89	0.20	0.21	0.25	0.23	5.79	6.23	8.24
NH_4_^+^ (μM)	0.13	0.11	0.13	0.12	0.14	0.15	0.09	0.08	0.03
Total N (μM)	3.68	4.36	0.40	0.41	0.48	0.46	6.33	6.90	8.89
N:P ratio	23.81	35.74	4.00	3.42	3.43	3.83	39.56	27.60	19.76
Sequencing statistics									
Total bp (Gb)	15.9	15.3	19.9	15.1	3.1	15.3	16.9	15.0	14.8
Mean read length (bp)	92	91.9	121.4	117.2	112.3	119.8	121.4	120.2	117.0
Mean GC (%)	40.7	41.1	38.6	41.4	40.4	40.6	41.3	41.1	45.9
Assembly statistics									
Total bp (Mb)	203	201	738.5	500.3	79.5	577.3	701.3	613.5	490.0
Mean GC (%)	38	39	38.5	35.7	35.5	38.6	38.9	40	42.2
Maximum contig length (Kb)	137	109	251	165	118	235	186	140	450
Contigs > 1 Kb	39,794	38,491	172,484	125,642	17,760	145,031	173,153	153,245	115,711
Contigs > 10 Kb	869	885	4648	2198	202	2360	2789	2556	1807

### Variability of environmental parameters

**Fig. 1:**
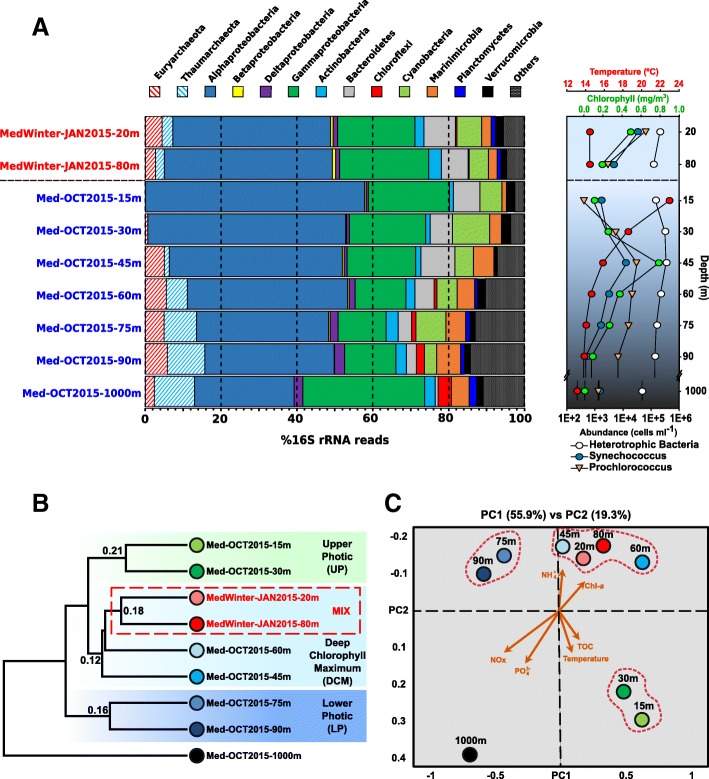
**a** Phylum-level composition based on 16S rRNA gene fragments (raw reads) of the different metagenomes. The phylum Proteobacteria was divided into its class-level classification. Only those groups with abundance values larger than 1% in any of the metagenomes are shown. Sample names in blue and red correspond to those collected in stratified and mixed water columns, respectively. On the right panel, a vertical profile of the temperature, chlorophyll-a, and abundance of heterotrophic bacteria, *Synechococcus* and *Prochlorococcus* cells is shown. The horizontal dashed line separates the mixed samples from the stratitied samples. **b** Dendrogram of metagenomic dataset raw sequence similarity. Samples highlighted with a red dotted line were collected in winter. Numbers at each node indicate similarity (1 would be 100% similarity) among the samples using raw reads. **c** Canonical correspondence analysis (CCA) between physicochemical parameters and read annotation similarity. Chl-a, chlorophyll-a; TOC, total organic carbon

Because of the prolonged physical isolation and accumulation of settled organic matter during the summer stratification period, bottom waters were richer in both dissolved and particulate inorganic nutrients (N and P) (Table 1 and Additional file 1: Figure S2). However, layers within the DCM were typically the richest of the photic zone, in terms of biomass accumulation. The surface water temperature for the samples taken during the stratification period was 22.9 °C, decreasing to 14.5 °C in deeper layers beyond the DCM. However, a similar temperature was found to be constant through the entire water column in winter (Table 1). Chlorophyll-a measurements indicated that the DCM occurred between depths of 40–60 m, just below the seasonal thermocline (Fig. 1a). Chlorophyll-a reached 0.8 mg m^−3^, almost one order of magnitude above those from surface waters and 100 times those from deep (1000 m) waters. Using flow cytometry, we measured the absolute numbers of planktonic picoprokaryotes for the whole water column (Fig. 1a). In the stratified period, the maximum of *Prochlorococcus* (nearly 3.2 × 10^4^ cells mL^−1^) and *Synechococcus* (1.35 × 10^4^ cells mL^−1^) were found in the DCM peak. These values were one to two orders of magnitude higher than those in surface waters. The distribution of *Prochlorococcus* cells was wider than that of *Synechococcus* (Fig. 1a), as previously described [25]. In the winter sampling, the seawater column was mixed, and no significant differences in temperature or any other physical or chemical features were observed (Table 1). We also measured the abundances of both heterotrophic and autotrophic prokaryotes, which were highest in the shallowest sample (20 m), though the relative abundance of active heterotrophic bacterioplankton was higher in the deepest sample (80 m) (Fig. 1a).

### Depth and seasonal variation of the prokaryotic community

Using the number of similar reads (> 95% identity) among metagenomes, we examined the relationship between the nine sequenced samples (Fig. 1b). The stratified samples were clustered by depth, with three main branches corresponding to (i) upper photic (UP, 15 and 30 m), (ii) DCM (45 and 60 m), and (iii) lower photic (LP, 75 and 90 m) layers. As shown in Fig. 1b, despite the different depths at which the mixed samples (MIX) were obtained (20 and 80 m), both clustered together within the group of DCM samples. As expected, the bathypelagic 1000 m sample appeared as an outgroup compared to all the photic zone samples. Independently, the canonical correspondence analysis (CCA) of the read annotations and environmental parameters confirmed the clustering of samples according to the depth and MIX with DCM (Fig. 1c). Inorganic nutrients (such as NOx and PO_4_^3−^) increased with depth, while ammonia correlated closely with chlorophyll-a, and total organic carbon (TOC) increased at the surface together with water temperature (Fig. 1c).

Measurements of Simpson’s Diversity Index at both genus and species levels clearly indicated that bacterial diversity increased continuously with depth only for the stratified season (Additional file 1: Figure S3). The 15 m sample was the least diverse of those from the photic zone and was markedly predominated by Pelagibacterales. At this depth, high light intensity and nutrient depletion generate conditions that can be considered extreme and that may result in the survival of only a few microbial taxa. In deeper waters, diversity increased with depth, particularly at the species level, reaching a maximum in our deepest sample at 1000 m (Additional file 1: Figure S3). The larger diversity of microbes in bathypelagic waters might correlate with a capacity to degrade or use a larger number of different substrates [26, 27]. While diversity increased with depth during stratification, the constant value in the winter samples was similar to that of the photic region at both the genus and species levels suggesting that the disturbances in the environment produce variations in the bacterial community that diminish the diversity in favor of more adapted species.

Together with diversity, other genomic parameters also varied with depth, such as the GC content (Table 1). The GC content was lowest at 15 m (*ca*. 38.6%) and highest at 1000 m (*ca*. 45.9%) while remaining relatively constant throughout the photic zone deeper than 15 m and in the winter samples (*ca*. 41%). The lower GC content observed in the near-surface stratified waters has been suggested to be a natural adaptation to reduce N demand in these environments with a severe depletion of bioavailable N pools [28].

Metagenome-derived 16S rRNA profiles revealed broad, depth-dependent variations in taxonomic ranges during stratification (Fig. 1a). Archaea, absent in the UP region, represent nearly 16% of the population at 90 m. In the DCM and LP samples, Euryarchaeota remained constant (*ca*. 5%), while Thaumarchaeota increased from 1% in the 45 m sample to 10% of all the rRNA reads in the 90 m sample. The abundance of Thaumarchaeota correlated with a sharp decrease in ammonia concentration, although the main increase in Thaumarchaeota occurred at 60 m, while ammonia concentrations showed the lowest values at 75 m (Fig. 1a and Table 1).

Whereas Actinobacteria, Bacteroidetes, Cyanobacteria, and Marinimicrobia were present in the whole water column, Deltaproteobacteria, Planctomycetes, Chloroflexi, and Acidobacteria had a much more restricted range, appearing only in deeper layers of the photic zone (Fig. 1a). Interestingly, Verrucomicrobia were present at all depths except in the 45 m sample. Using finer-scale taxonomic classification of the 16S rRNA sequences, we found that UP (15 and 30 m) Verrucomicrobia belonged to *Puniceicoccaceae*, whereas the members of *Verrucomicrobiaceae* were predominantly found below the DCM (Additional file 2: Table S1). Although the results clearly reveal ecologically distinct lineages that occupy different niches, we still know very little about the ecophysiology of these Verrucomicrobia lineages in seawater. The proportion of 16S rRNA gene reads assigned to unclassified bacteria also increased with depth, from 3% at 15 m to more than 10% at 90 m, indicating that a significant fraction of the microbes at the subsurface is still uncharacterized.

Furthermore, our results indicate no significant changes in prokaryotic diversity during seasonal fluctuations as long as the entire water column is taken into account rather than only a single depth. A homogeneous community distribution similar to the DCM and LP samples was observed in the MIX samples. For example, it has been suggested that through using pyrosequencing 16S rRNA gene PCR amplicons in a surface sample (3 m depth) in the northwestern Mediterranean Sea, Thaumarchaeota MGI and Euryarchaeota MGII-B populations were more abundant during winter [29]. However, our results show that archaea were always present and abundant throughout the water column during the winter but were almost absent in the UP region during the stratification. Similar observation was made using metatranscriptomes from the stratified water column in the Gulf of Aqaba/Eilat [30]. In the same way as the Planctomycetes or Chloroflexi that only appeared below the DCM (Fig. 1a). Even at lower taxonomic ranks, the distribution was similar, except for some specific families such as *Sphingomonadaceae*, *Alteromonadaceae*, and *Pseudomonadaceae*, that predominantly increased in the deeper layers during the winter (Additional file 2: Table S1). This finding highlights the importance of collecting samples at different depths in the water column when comparing seasonal variations and has important ramifications for global marine studies that most often take samples only from the surface or, at most, from one single subsurface photic zone depth.

### Metagenome-assembled genomes

The broad organismal distributions detected by 16S rRNA genes or raw sequence annotation methods described above, however, do not shed light on the more subtle but ecologically significant variations in community structure or metabolic function that likely occur at the finer levels of diversity, such as ecotypes, or even clonal lineages, within species [31–34]. To investigate the distribution of the major ecotypes present in the water column, we used stringent recruitment of metagenomic reads for assemblies of locally predominant metagenome-assembled genomes (MAGs). We have used the same approach with several metagenomes obtained closer to the sampling site [35–37].

Overall, using a combination of different parameters, such as GC content, metagenomics read coverage, and tetranucleotide frequencies, we have retrieved new MAGs belonging to phyla for which we obtained more than 5 Mb of assembled contigs (Additional file 3: Table S2 and Additional file 1: Figure S4). These genomes were classified phylogenomically using concatenated sequences of conserved proteins (Additional file 1: Figures S5–S12). In the end, we were able to obtain 94 novel MAGs.

In general, genome assembly improved proportionally with the abundance of the phylum. However, we found that genomes of representatives from Bacteroidetes, Actinobacteria, and Acidobacteria assembled better than expected based on their abundance by 16S rRNA gene fragments recovered (Additional file 1: Figure S4 and Additional file 2: Table S1). On the other hand, Cyanobacteria, Thaumarchaeota, and Pelagibacterales assembled much more poorly. Both picocyanobacteria and Pelagibacterales are known to possess enormous intra-species diversity [38], which might be the reason why the assembly for these two major components of the bacterioplankton was very poor.

### Relative abundance of the prokaryotic community

To examine the patterns of relative abundance and diversity of the microbial communities among the metagenomes, we performed metagenomic recruitments of the reads over the MAGs as well as several reference genomes from public databases, taking into account only the reads that match the genomes with a similarity ≥ 99% in our metagenomic samples, thus representing finer levels of diversity. To simplify, we set a threshold of three reads per kilobase of genome and gigabase of metagenome (RPKG) in at least one sample to establish the presence of these genomes.

#### Fine taxonomic profile (eurybathic and stenobathic)

Additional file 1: Figure S13 shows the recruitment of MAGs obtained from the stratified metagenomic samples of this study (from MED-G01 to MED-G44), MAGs from other metagenomic samples previously described from the same site (from MED-G45 to MED-G94), and several selected genomes of marine isolates sourced from public databases (21 recruited more than 3 RPKG and are shown in the figure). It is remarkable how uneven the recruitment depth profile was for the vast majority of the microbial genomes, particularly considering that all the samples were collected on the same day (except the 1000 m). All the genomes recruited much more at one single specific depth and most (*ca*. 70%) recruited only from metagenomes sampled at either one or two consecutive depths (stenobathic). This result indicates that the distribution of most of these microbes only extends over a 30-m-thick layer within the *ca*. 100 m deep photic zone. Only one of the photic zone genomes, Sphingomonadaceae MED-G03, recruited in the 1000 m sample. This genome actually recruited more at this depth and it may be the only truly eurybathic microbe among the ones assembled here. The actinobacterial genomes seemed to be the next most eurybathic, and although they always appeared to be more prevalent at a single depth, they were detectable at four depths, with the lone exception of the single cell genome SCGC−AAA015−M09 [39] (only found at 15 and 30 m). Alphaproteobacteria (with the exception of Pelagibacterales), such as most Bacteroidetes and Gammaproteobacteria, were only detected at one or two depths. Most microbes were preferentially found at the UP or DCM depths except for some archaea. For example, members of the MGI Thaumarchaeota and some groups of Euryarchaea appear to prefer the LP (Additional file 1: Figure S13). *Ca*. Nitrosopelagicus brevis [40] and Nitrosopumilus MED-G94 possess the complete cluster for ammonia oxidation and are expected to increase with depth due to the much higher availability of its major substrate (ammonia). Moreover, their abundance in this region is also correlated with the light intensity attenuation in deeper waters due to the ammonia oxidation photoinhibition [41]. We utilized the relatively large collections of available pure culture genomes of picocyanobacteria and used the ones that had contigs with high similarity (close to 100%) as proxies of local genomes. *Synechococcus* MAGs were practically identical (> 99.2% average nucleotide identity [ANI]) to the isolated genomes, whereas *Prochlorococcus* MAGs where closely related but not identical (97–98% ANI) (Additional file 1: Figure S8). Recruitments of cultured picocyanobacteria occurred over a range similar to the locally assembled genomes, and again, the clear depth preferences were apparent. In Cyanobacteria, there are low/high light-adapted ecotypes, as has been repeatedly described in several oceanic regions [23, 42]. The first 45 m were dominated by the HLI clade (the pure culture *Prochlorococcus* MED4 and the MAG Prochlorococcus MED-G72) with a peak in abundance at approximately 30 m, which then decreased below this depth when clade LLI (*Prochlorococcus* NATL1A and Prochlorococcus MED-G73) appeared. On the other hand, *Synechococcus* genomes were not detected deeper than 30 m (Additional file 1: Figure S13).

#### Seasonal dynamics of the community structure

**Fig. 2:**
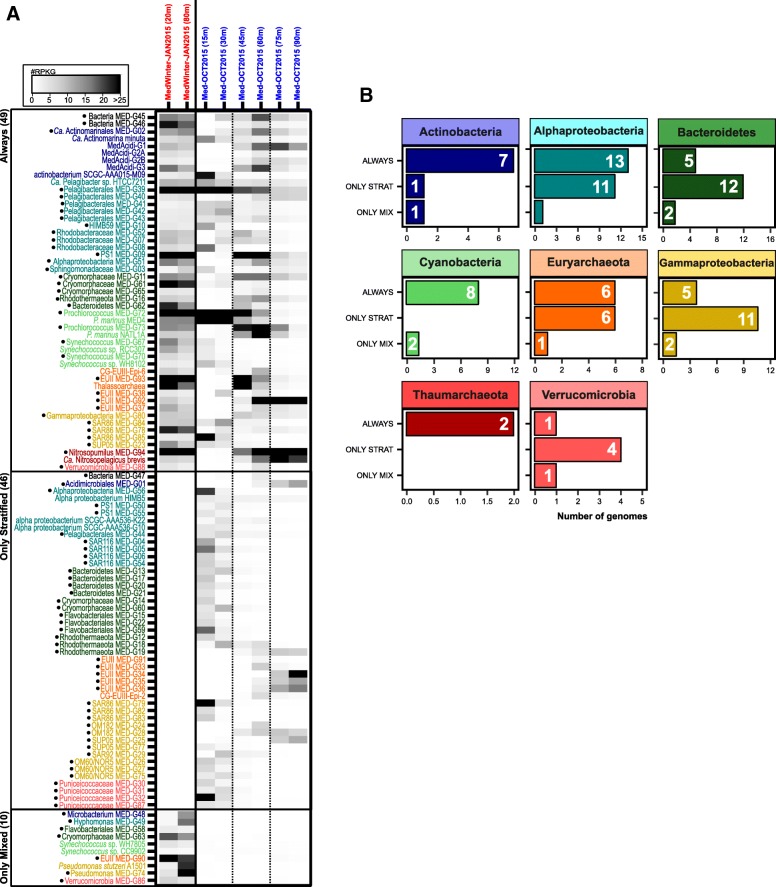
**a** Metagenomic recruitment (≥ 99% identity) of genomes that recruited at least three reads per kilobase of genome per gigabase of metagenome (RPKGs) in any of the samples is shown in the left panel. Genomes are sorted and colored according to their taxonomy. Genomes with a dot were reconstructed in this work. **b** Number of genomes (*x*-axis) for each taxon that are present always, only during stratification, and only during the mixing event (winter)

**Fig. 3:**
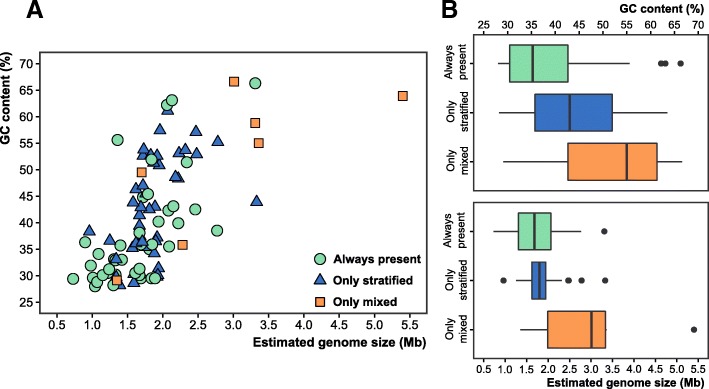
**a** Two-dimensional plot comparing GC content and genome size (estimated by CheckM, see the “Methods” section) for all the reconstructed genomes that appear in Fig. 2. **b** Box plots of GC content (top) and genome size (bottom). Genomes are classified and colored depending on whether they appear always in the water column (green), only during stratification (blue), or only in winter during the mixing period (orange)

To analyze the impact of the strong winter convection on the community, we included two more samples obtained in winter (January 2015) at 20 and 80 m depth, during the period when the water column is fully mixed. Using the previous criteria, we found that despite the strong variability in the physicochemical parameters (light, temperature, and nutrients), 47% of the genomes were found in both the mixed and the stratified periods. In fact, based on relative abundances, microbes that were found only in small ranges of 15 to 30 m deep during the stratified period were present at similar values at both depths (20 and 80 m) when the water column was mixed (Fig. 2a). Among the groups that were always present, 21 out of 49 (43%) were Alphaproteobacteria and Cyanobacteria (mainly the SAR11 clade and *Synechococcus*, respectively). Some less abundant, but nevertheless resistant, taxa (always present) included members of the Actinobacteria families *Acidimicrobiaceae* (MedAcidi-G1, G2A, G2B, G3) and *Ca*. Actinomarinaceae (*Ca*. Actinomarina minuta), three SAR86 clade genomes within Gammaproteobacteria and the Bacteroidetes family *Flavobacteriaceae* (Fig. 2a, b). Most of these groups represent ubiquitous and abundant heterotrophic microbes characterized by a small genome size and low GC content (and likely a small cell size and more efficient absorption of nutrients) (Fig. 3), highly adapted to oligotrophic environments by metabolic streamlining (*K*-strategists) [43–47]. This persistence could be due also to their ability to use organic matter along with light energy through light-dependent proton pumps (rhodopsins). It seems likely that these capabilities allow for better adaptation to overcome the environmental disturbances produced during winter mixing and subsequent phytoplankton blooms. Unlike what the literature has described so far, we measured a temporal persistence of some taxa previously considered sporadic or rare, such as archaea [22, 29, 48, 49]. Although these taxa only recruit below the photic zone during the stratified period, members of the ammonia-oxidizing group I Thaumarchaeota and groups II and III Euryarchaeota were always found to be present in the water column.

Conversely, most of the microbes that we found only during winter could be considered opportunistic (*r*-strategists or bloomers) and are microbes that grow rapidly, taking advantage of the sporadic inputs of organic matter that appear in the environment. However, although they can be easily retrieved in pure culture, they are usually rare in seawater. These microbes could be associated with the decay of the phytoplankton blooms and higher nutrient levels [50, 51]. We were able to assemble seven genomes only found in winter that were classified within the Actinobacteria, Gammaproteobacteria, Verrucomicrobia, Bacteroidetes, and Euryarchaeota phyla (Fig. 2). As was previously shown in Fig. 3, these genomes were characterized by having a large estimated genome size (> 3.0 Mb) and a high GC content (> 50%). Additionally, these genomes also possess multiple clusters for degrading a wide range of substrates as well as genes responsible for flagellum biogenesis and motility, which are typical metabolic properties of heterotrophic bacterial communities associated with these phytoplankton blooms [50].

Remarkably, 46 MAGs were only present during stratification, being totally absent in winter (Fig. 2). Many of these MAGs were members of the phyla Bacteroidetes (12 genomes), Verrucomicrobia (4 genomes), members of the SAR116 clade of the Alphaproteobacteria (Additional file 1: Figure S6) and the OM60/NOR5 clade within the class Gammaproteobacteria (Additional file 1: Figure S10). The vast majority of these genomes were found to be restricted to the UP layer. However, members of MGII archaea, OM182, and SUP05 clades of Gammaproteobacteria, that also disappear in winter, came from deeper layers (DCM and LP). A seasonal analysis carried out in surface waters of Blanes [22], Bermuda [18], and the North Sea [52] showed variations in the concentration of members of these clades throughout the year, with a maximum in mid-summer and a near absence in winter when the water column was mixed and which were mostly limited to surface waters [22, 52, 53] in agreement with our data.

### Depth stratification of rhodopsins

Rhodopsins have been shown to be among the most widespread genes in the photic zone worldwide [54–56]. They are very diverse and are distributed throughout most taxa. We found 28 rhodopsin genes in both winter samples, but just one gene recruited only in winter and not during stratification. This rhodopsin (within the MAG Verrucomicrobia MED-G86) was analyzed in detail (see below) and belonged to the Planctomycetes-Verrucomicrobia-Chlamydiae (PVC) superphylum. In the end, a total of 105 out of 196 rhodopsin genes (53%) recruited only during stratification, 46% in both, and just 1 rhodopsin gene only in winter.

**Fig. 4:**
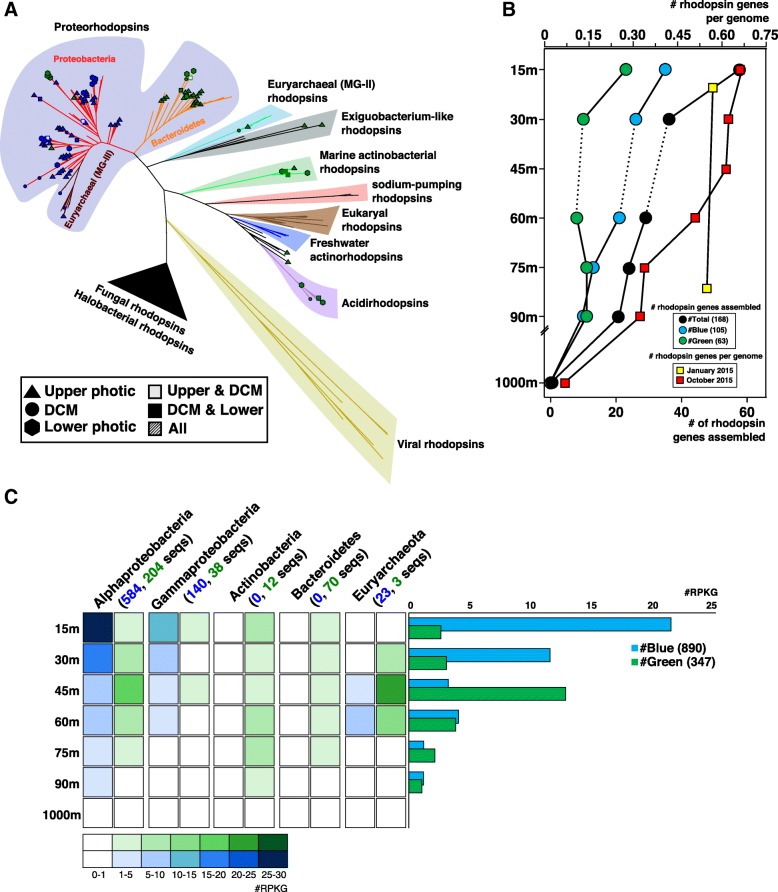
**a** Phylogenetic tree of rhodopsin genes detected in the different depth metagenomes. Sequences obtained in this work are represented according to depth and color absorption (green or blue). **b** Number of assembled rhodopsin genes at different depths. Reads annotated as rhodopsin normalized by the number and length of recA+radA genes (estimated rhodopsin genes per genome) are indicated by red squares. **c** Recruitment of all rhodopsins including those obtained in this work together with the publicly available in the MicRhoDE database. Left panel, rhodopsins classified according to their taxa of origin. Right panel, average number of blue/green rhodopsins for each depth. Numbers within the brackets indicate the number of rhodopsins

We evaluated the numbers of rhodopsins among the individual reads and calculated their frequency per genome, normalizing them by the number of single copy housekeeping genes (*rec*A and *rad*A) and by their gene length (Fig. 4b). The total numbers of rhodopsin-assigned reads were clearly correlated to light intensity, with a maximum at 15 m, where ca. 65% of the genomes contain a rhodopsin, which then decreased with depth. This result is different from the situation in the permanently stratified central North Pacific, where the maximum was found at the DCM [56]. Conversely, for winter samples, the number of rhodopsins was similar regardless of depth throughout the water column, as was expected due to the mixing event.

We assembled 168 rhodopsin genes throughout the stratified water column. All of the genes were classified at least at the phylum level based on the flanking genes (Fig. 4a). The phylogenetic analysis revealed a large diversity of this gene family, and at least 11 major evolutionary lineages were detected. All the assembled rhodopsin genes clustered with previously described groups, indicating that surveys may have achieved saturation with the extant diversity of rhodopsins, at least in the oligotrophic ocean photic zone. Rhodopsin sequences clustered primarily by phylum, with the exception of euryarchaeal rhodopsins as previously reported [36, 57]. Within the proteorhodopsin cluster, we clearly differentiated a separate cluster including only Bacteroidetes sequences (Fig. 4a). Within the clusters, rhodopsin sequences were also grouped by depth, with many branches containing only upper or lower photic zone varieties. This result confirms the stenobathic character of most groups at the finer level of diversity resolution.

Rhodopsin genes from our metagenomic assemblies and from the MicRhoDE database [58] were used to recruit reads from the different depths (Fig. 4c). We observed no correlation between the predicted absorption spectrum (blue versus green light) of the rhodopsins and of the depth from which they recruited the most reads. In contrast, we did see a consistent pattern of correlation between the absorption spectrum and the phylogenetic affiliation of the host genome; Bacteroidetes and Actinobacteria all carry green rhodopsins, while Proteobacteria largely have the blue variety. The findings suggest, as previously reported [55, 59], that the spectral tuning of rhodopsins may not be related to depth adaptation but tend to be associated with the classification of the microbe instead.

Interestingly, within the MAG Verrucomicrobia MED-G86 (3.19 Mb and 55% GC content), we found the unique rhodopsin that recruited only in the MIX samples but not in the stratified. This is the first marine rhodopsin that clustered together with a novel clade of freshwater rhodopsins [60, 61] affiliated closely with the Exiguobacterium rhodopsins [62], confirming that this group is a characteristic of the Planctomycetes-Verrucomicrobia PVC superphylum (Additional file 1: Figure S14). Since this is the first marine representative, we searched in the *Tara* Oceans assembled contigs > 5 Kb for similar members in this group. Eight genomic fragments containing rhodopsin that clustered with this novel branch were retrieved (Additional file 1: Figure S14). It is remarkable that although two sequences came from the Mediterranean Sea (stations 009 and 030), the remaining six came from the North and South Pacific Oceans (stations 093, 094, 102, 109, 128, and 136). Furthermore, within the novel clade, we found another rhodopsin subcluster formed only with *Tara* sequences. However, the contigs that contained these sequences differed from the others in GC content, with low values between 35 and 40% instead of the high GC values found in Verrucomicrobia MED-G86 and the freshwater MAGs (Additional file 1: Figure S14). Unfortunately, we failed to classify taxonomically these contigs due to the ambiguous annotation of their proteins (proteins were annotated either as Verrucomicrobia or Planctomycetes).

### Functional analysis of the stratified and mixed water column

**Fig. 5:**
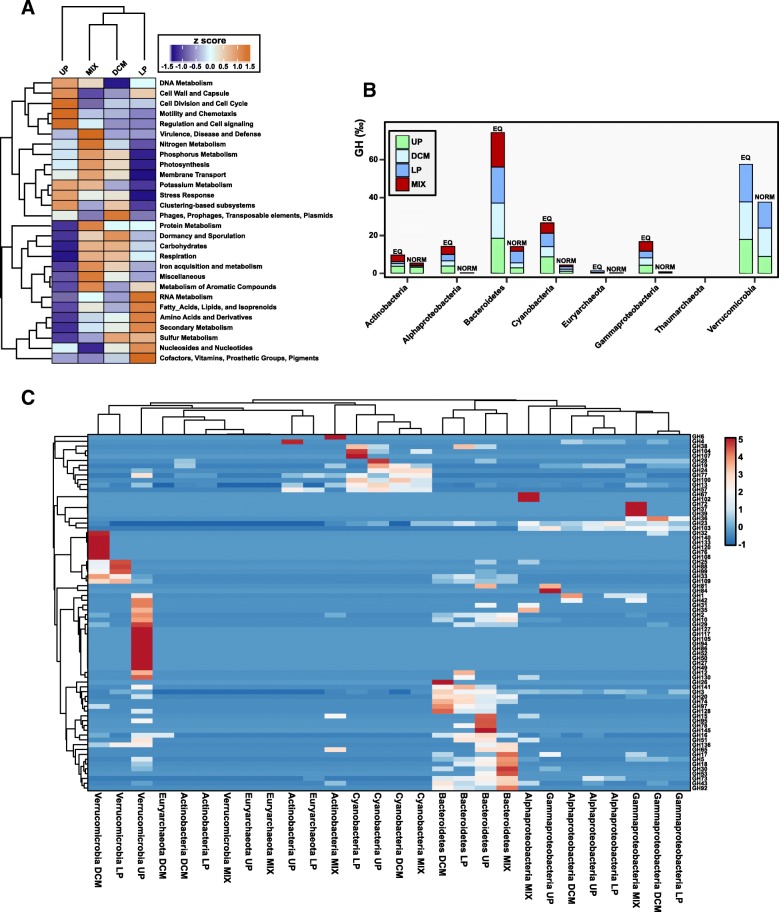
**a** SEED subsystems-based heatmap using the assembled coding sequences coming from contigs > 1 Kb. Proteins were grouped by depth (stratified samples UP, DCM, and LP) and season (MIX). For each one of the SEED categories, values were normalized by their standard score (*z*-score). **b** Number of glycoside hydrolases (GHs) detected in all the contigs > 5 Kb assigned the different phyla using the Carbohydrate-Active enZYmes (CAZy) database. EQ, number of GH per 1000 genes analyzed. NORM, number of GH per 1000 genes normalized by the percentage of 16S rRNA reads. **c** Heatmap of the different GH families. Abundance of GH was clustered by phylum and depth of the samples

To make a functional characterization of the microbial community associated with the metagenomes, we used the assembled coding sequences collected from contigs > 1 Kb against the SEED Subsystems database [63]. Clustering of level 1 subsystems (Fig. 5a) revealed a marked discontinuity between UP samples and the other samples, indicating unique characteristics of surface waters, while once again, the DCM and MIX samples clustered together, demonstrating that they are similar on a functional level, in concordance with the taxonomic clustering based on PCA (Fig. 1a). After comparing the number of proteins assigned to each subsystem, we found significant differences (based on the standard deviation among samples) mainly involving carbohydrates, membrane transport, and motility and chemotaxis.

#### Carbohydrates

To study the taxonomical and in-depth distribution of the genes encoding the glycoside hydrolases (GH) family of enzymes, which are involved in the breakdown of complex sugars, we compared all the proteins extracted from contigs larger than 5 Kb assigned to the phyla Actinobacteria, Bacteroidetes, Euryarchaeota, Thaumarchaeota, and Verrucomicrobia, as well as the classes Alphaproteobacteria and Gammaproteobacteria (all of which comprised more than 85% of the metagenomic 16S rRNA gene reads for all the samples), against the CAZy database [64].

The phylogenetic distribution of the CAZy genes was analyzed, considering the number of GH per 1000 genes (EQ) and the abundance normalized by the percentage of 16S rRNA gene reads of each group (NORM). Figure 5b shows that the abundance varied across bacterial phyla, and most of the genes were mainly derived from Verrucomicrobia, Bacteroidetes, and Cyanobacteria. Thaumarchaeota showed no GHs within the contigs, demonstrating an inability to degrade complex polysaccharides, as was expected from chemolithoautotrophs [40, 65]. Notably, within each group, the number of GH genes was similar at the different layers of the water column, although the types of GHs were different, suggesting specialization in the degradation of different polysaccharides that is likely connected with specific groups of algae or particles.

As expected [16, 66], Bacteroidetes was the group with more enzymes (74.3 GHs/1000 genes) (Fig. 5b). Clustering based on the abundance of the samples showed that Bacteroidetes from DCM and LP samples grouped together and separated from UP, which in turn was close to the MIX samples. We found some predominant GH families in winter, the two most abundant were endo-β-1,3-glucanases of the families GH5 and GH17, and a GH30 exo-β-1,6-glucanase (Fig. 5c). These enzymes are involved in the cleavage of the main storage polysaccharide (β-glucan) present in brown algae (laminarin) and in diatoms (chrysolaminarin) [67].

Verrucomicrobia represented the second group that included the largest number of GH genes, with 54.3 GHs/1000 genes analyzed. The results showed that the majority of the GHs present in Verrucomicrobia were different from Bacteroidetes, indicating that members of these phyla may be utilizing different carbohydrate substrates (Fig. 5c). As with Bacteroidetes, we found that the number of GH families was higher in Verrucomicrobia from UP than in DCM and LP. This result suggests that deeper Verrucomicrobia shows less variability in degrading potential substrates. Specifically, we found an overrepresentation of alpha- and beta-galactosidases, xylanases, fucosidases, agarases, and endoglucanases in UP Verrucomicrobia. The most abundant family at all depths was GH109, with the only known activity being that of an α-*N*-acetylgalactosaminidase that might degrade the peptidoglycan of the cell walls [68].

Remarkably, Cyanobacteria was the third group with a higher number of GHs (Fig. 5b). Unlike the previous cases, the type of GH family was similar in all the samples and was associated with amylose degradation (GH13 and GH57—α-amylase; GH77—amylomaltase). These three GH families were also found in Actinobacteria (UP and MIX) and in Euryarchaeota (LP), which shared the same metabolic potential. Additionally, clustering showed that Cyanobacteria from DCM and MIX shared similar values for the families GH19 and GH24, both with chitinase/lysozyme activities. Thus, the degradation of complex sugars (i.e., amylose or chitin) increases their capability to obtain organic carbon. It has been described that both Cyanobacteria (*Prochlorococcus* and *Synechococcus*) also harbor genes that encode a wide number of amino acid, peptide, and sugar transporters [69–71], which allow them to uptake organic compounds, that together with the ability to obtain energy using the sunlight (mixotrophy) seems to be present in all the marine *Synechococcus* and *Prochlorococcus*, and globally distributed in the photic zone of the oceans [71]. Recently, it has been shown that mixotrophy can increase the viability of *Prochlorococcus marinus* during extended periods of darkness, due to the coculture with a marine copiotroph, *Alteromonas macleodii*, which may be supplying organic compounds to *Prochlorococcus*. Our results, together with previous studies, highlight the mixotrophic nature of marine picocyanobacteria, as several glycoside hydrolases are encoded in their genomes.

Although Alpha- and Gammaproteobacteria comprised > 50% of the prokaryotic community (based on the metagenomic 16S rRNA gene reads), they possessed very low numbers of GHs (14.1 and 16.6 GHs/1000 genes, respectively), indicating a different functional role in the marine ecosystem.

#### Membrane transport

We analyzed the abundance of genes affiliated with membrane transport using KEGG modules. PCA analysis was performed to determine the clustering of the samples (Additional file 1: Figure S15). The results showed that the mixed samples clustered together and separated from the stratified samples, which, in turn, were also clustered by depth for UP and LP samples, while the DCM samples showed a more dispersed distribution. In terms of nutrient acquisition, we found transport systems (ATP-binding cassettes and phosphotransferases) related to iron, phosphonate, polyamines (putrescine/spermidine), oligopeptides, and sugars, and several heavy metal resistances such as the cobalt-zinc-cadmium (CzcA) efflux system, which are typically components of the flexible genome of some bloomers [72], were enriched in the winter-mixed samples. This wide variety of transporters might allow for uptake and use of a large quantity of phytoplankton-derived compounds. During the stratification, in the lower layers of the water column beyond the DCM, in addition to putative specific transporters for Archaea (A2 holin family), we found a higher proportion of ABC di/oligopeptide transporters. TonB-dependent transporter proteins are relatively abundant particularly in UP. These transporters allow the uptake of scarce resources (i.e., iron complexes and other nutrients [73]) from nutrient-limiting environments such as surface layers due to their high affinity. Choline and betaine uptake proteins that play an important role in bacterial osmoregulation and stress tolerance were also abundant in the UP [74]. For instance, the SAR11 clade, which based on 16S rRNA data is the most abundant here, was enriched in these transporters that are highly active based on transcriptome data [75].

#### Motility and chemotaxis

Motility is another adaptation that differentiates copiotrophs from oligotrophs [45]. Despite that UP presents the highest value in abundance of genes related to the SEED category “motility and chemotaxis,” this region is dominated by members of the SAR11 clade, which have no genes encoding for flagellar synthesis or chemotaxis proteins. Manual inspection of the contigs including these proteins revealed an enrichment in high GC-content microbes mainly from Alpha- (Sphingomonadadales and SAR116) and Gammaproteobacteria (Oceanospirillales) classes. These genomes probably assembled better due to the lower intra-species diversity. Remarkably, within the group of MIX samples, bacteria from MedWinter-JAN2015-80m exhibited a significantly large number of genes involved in chemotaxis but not for biosynthesis of the flagella in comparison with all the other samples (Additional file 4: Table S3). These results suggest that a mechanism to sense and respond to the chemicals likely released by phytoplankton is an important competitive advantage for opportunistic bacteria during winter when the access to nutrients increases. Other functions reflected the interaction with phytoplankton blooms, for example, the inclusion of modules involved in the detoxification of reactive oxygen species (ROS) since phytoplankton are the most important source of ROS in the water column [76] or peptidases to process phytoplankton-derived organic matter [77]. Many studies have demonstrated that there is a mutualistic or parasitic interaction between bacteria and phytoplankton [50].

## Conclusions

**Fig. 6:**
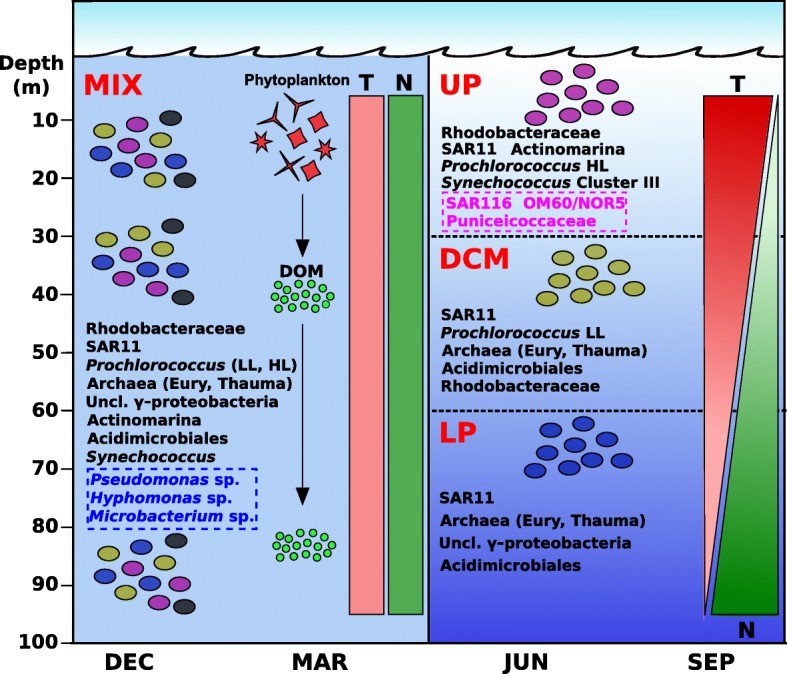
A graphic summarizing the prokaryotic community dynamics in both stratified and winter seasons. In winter (left panel), physicochemical parameters like temperature (T, in red) and nitrogen (N, in green) do not vary with depth throughout the photic zone, and microorganisms extend over the mixed water column. In summer and part of the autumn (right panel), the stratification of the water column is mainly due to the differences in temperature between surface and deep waters. During this season, three major regions divide the photic zone: upper photic (UP), deep chlorophyll maximum (DCM), and lower photic (LP), and therefore, most of the microorganisms are restricted to one specific region. Persistent microorganisms (in black) are always present, independently of the season. During the mixed season, these microbes span over the water column, while during summer, they inhabit preferentially one or more regions of the photic zone. During winter, some *r*-strategist microbes (in blue) can grow easily due to the upwelling of nutrients from mesopelagic waters and the release of dissolved organic matter from phytoplankton blooms. Conversely, some *K*-strategist microbes (in pink) only grow in summer, during the stratification event. LL, low light; HL, high light

The photic zone of aquatic habitats is subjected to several strong gradients of environmental parameters. In the Mediterranean, similar to in most temperate seas, thermal stratification appears only during warmer periods, typically from May to November, while in winter, the water column is mixed and the gradient of nutrients disappears. Although there is abundant information about the prokaryotic community composition during the stratified and mixed periods, most previous works derive from 16S rRNA-related techniques (such as FISH or barcoding approaches). These approaches do not have enough resolution at the species or ecotype levels. Here, we have used metagenome recruitment as an alternative to detect specific MAGs and some previously described genomes to assess the distribution of specific microbial genomes in a fine depth profile (every 15 m) from an stratified and also in a mixed water column during winter. We found major depth-associated shifts in the community structure during the stratified period and that, particularly at the level of fine variation, most microbes had a distribution covering only a ca. 30-m-thick layer of seawater and were stenobathic. During the stratified period, it is necessary to consider the vertical distribution as the major element instead of comparing single depth samples. Thus, we found that some microbes previously considered rare or seasonal (such as archaea or Actinobacteria) are actually resistant to seasonal variations. These microbes generally live in deeper layers within the photic zone during the stratification period (Fig. 6). Our results also indicate a strong specialization not only at the taxonomic level but also at the functional level, even within the different clades, for the manipulation and uptake of specific polysaccharides and likely for the succession of different bloom events. This finding has important ramifications for global marine studies that most often take samples only at the surface or, at most, from one single subsurface photic zone. Moreover, the description of seasonal dynamics within the water column has important implications in the analysis and response to future alterations in the water conditions due to climate change. Mainly, an increase of the seawater temperature will produce a change of the physical mixing dynamics, where the upwelling of nutrients from the deeper layers to the surface will be prevented, reshaping the microbial community structure. As a result, this will have direct consequences on microbial metabolism, which will modify the marine global biogeochemical cycles (mostly carbon and nitrogen) [78].

## Methods

### Sampling, sequencing, assembly, and annotation

Six samples from different depths were taken for metagenomic analyses on 15 October 2015 at a single site from the western Mediterranean (37.35361° N, 0.286194° W), at approximately 60 nautical miles off the coast of Alicante, Spain, from the research vessel “García del Cid.” These seawater samples (200 L each) were collected from the uppermost 100 m at 15 m intervals using a hose attached to a CTD (Seabird) connected to a water pump, to directly transfer seawater from the selected depth to the filtration system, and thus minimize sample storage time and potential bottle effects (Additional file 1: Figure S1). Each sample was filtered in less than 30 min. Another sample from a depth of 1000 m was taken the next day (16 October) in two casts (100 L each) using the CTD rosette. Two more samples were collected on 27 January 2015, at 20 and 80 m depth, at 20 nautical miles off the coast of Alicante (38.068° N, 0.232° W).

All seawater samples were sequentially filtered on board through 20, 5, and 0.22 μm pore size polycarbonate filters (Millipore). All filters were immediately frozen on dry ice and stored at − 80 °C until processing. DNA extraction was performed from the 0.22 μm filter as previously described [79]. Metagenomes were sequenced using Illumina Hiseq-4000 (150 bp, paired-end read) (Macrogen, Republic of Korea). Individual metagenomes were assembled using IDBA-UD [80]. The resulting genes on the assembled contigs were predicted using Prodigal [81]. tRNA and rRNA genes were predicted using tRNAscan-SE [82], ssu-align [83], and meta-RNA [84]. Predicted protein sequences were compared against NCBI NR databases using USEARCH6 [85] and against COG [86] and TIGFRAM [87] using HMMscan [88] for taxonomic and functional annotation. GC content and richness in each sample were calculated using the gecee program from the EMBOSS package [89] and MEGAN6 Community Edition [90], respectively.

### Vertical profiles and chemical features

Vertical profiles of several physical, chemical, and biological variables were determined in situ using a Seabird SBE 19 multiprobe profiler coupled to several fluorometric probes. Variables measured were temperature (SBE), dissolved oxygen (SBE43), pH (SBE27), chlorophyll-a concentration (WETStar), phycoerythrin (Seapoint) and phycocyanin (Turner) fluorescence, turbidity (Seapoint), and chromophoric dissolved organic matter (cDOM) concentration (Wetlabs). Other chemical variables, inorganic soluble forms of nitrogen (NOx and ammonium), and phosphorus (soluble reactive phosphorus), as well as total nitrogen (TN) and total phosphorus (TP), were performed following standard methods for water analyses [91]. Total organic carbon (TOC) was determined on a Shimadzu TOC-VCSN Analyser. Quantitative determination of chlorophyll-a concentrations was determined by HPLC after extraction in acetone following [92].

### Microbial counts

The abundance of heterotrophic and autotrophic picoplankton (*Synechococcus* and *Prochlorococcus*) were determined using a Coulter Cytomics FC500 flow cytometer (Brea, California, USA) equipped with two different lasers, an argon laser (488 nm excitation) and a red-emitting diode (635 nm excitation), and five detectors for fluorescent emission (FL1–FL5). Quantitative counts of heterotrophic bacterioplankton and its relative DNA content (HDNA versus LDNA cells, as a relative measure of activity) [93] were performed after cell DNA staining with Sybr Green I (Sigma-Aldrich, Missouri, USA) following [94]. Using the green fluorescence of Sybr Green I, the argon laser allowed detecting the cells with the FL1 detector (525 nm). The abundance of autotrophic picoplankton was determined by combining the argon laser and the red diode with the red fluorescence of chlorophyll-a and phycobiliproteins, using the FL4 detector for the identification of the populations of *Synechococcus* and *Prochlorococcus*. Their cells were differentiated by both their fluorometric signature and size features. Cytometric parameter settings were FSC (550), SSC (390), FL1 (600), FL2 (670), FL3 (670), FL4 (620), and FL5 (700). Analyses were run for 160 s at the highest possible single flow rate (128 μL min^−1^). Abundance of each population was calculated according to the formula: *N* = (*n* × 1000)/(*q* × *t*), where *q* is the flow rate (microliter per minute), *t* is the duration (minutes) of the acquisition, *n* is the number of events counted by the flow cytometer, and *N* is the number of cells per milliliter. Data were collected using the Beckman Coulter software for acquisition “CXP Version 2.2 Acquisition,” and the analysis of the data was performed using the Beckman Coulter software for analysis “CXP Version 2.2 Analysis.”

### Phylogenetic classification

A non-redundant version of the RDP database [95] was prepared by clustering all available 16S/18S rRNA gene reads (*ca*. 2.3 million) into approximately 800,000 clusters at 90% identity level using UCLUST [85]. This database was used to identify candidate 16S/18S rRNA gene sequences in the raw metagenomes (subsets of 10 million reads). Using USEARCH [85], sequences that matched this database (*E* value < 10^−5^) were considered potential 16S rRNA gene fragments. These candidates were then aligned to archaeal, bacterial, and eukaryal 16S/18S rRNA HMM models [96] using ssu-align to identify true sequences [83]. Final 16S/18S rRNA sequences were compared to the entire RDP database and classified into a high-level taxon if the sequence identity was ≥ 80% and the alignment length ≥ 90 bp. Sequences failing these thresholds were discarded.

### Binning and genome reconstruction

Assembled contigs longer than 10 Kb were assigned a high-level taxon classification if > 50% of the genes shared the same taxonomy. The rest of the contigs were grouped together as unclassified. To bin the contigs into MAGs, their taxonomic affiliation (including unclassified group) was used together with the principal component analysis of tetranucleotide frequencies, GC content, and coverage values within the metagenomes collected in this work, together with those described in [36, 37]. Tetranucleotide frequencies were computed using wordfreq program in the EMBOSS package [89]. The principal component analysis was performed using the FactoMineR package [97] in R. Completeness of the MAGs was estimated by comparison against two different universal gene sets, one with 35 genes [98] and another with 111 genes [99], and with CheckM, which also provides the degree of contamination [100]. In order to improve the completeness and remove the redundancy, a second assembly step was performed combining the genomic fragments with the short paired-end Illumina reads of the metagenomes from which they were assembled. For each genome, we used the BWA aligner [101] with default parameters to retrieve the short paired reads that mapped onto the contigs. These reads were then pooled and assembled together with the contigs using SPAdes [102].

### Metagenomic read recruitments

The genomes of known marine microbes together with the genomes reconstructed in this study were used to recruit reads from our metagenomic datasets using BLASTN [103], using a cutoff of 99% nucleotide identity over a minimum alignment length of 50 nucleotides. Genomes that recruited less than three reads per kilobase of genome per gigabase of metagenome (RPKG) were discarded.

### Phylogenomic trees of the reconstructed genomes

Phylogenomic analysis was used to classify and identify the closest relatives for all the reconstructed genomes. Using HMMscan, we aligned the sequences against the COG database. Shared proteins were concatenated and aligned using Kalign [104]. A maximum-likelihood tree was then constructed using MEGA 7.0 [105] with the following parameters: Jones-Taylor-Thornton model, gamma distribution with five discrete categories, and 100 bootstraps. Positions with less than 80% site coverage were eliminated.

### Metagenomic cross-comparisons

Two different approaches were used to compare similarities between metagenomic samples. First, a reciprocal global alignment of the short Illumina reads (in subsets of 2 million reads ≥ 50 bp) at ≥ 95% identity was performed using USEARCH6. The results of the comparison were then clustered with the hclust package in R using a euclidean distance matrix. In a second approach, subsets of 20 million reads ≥ 50 bp (where applicable) were taxonomically classified against the NR database using DIAMOND [106] with a minimum of 50% identity and 50% alignment. The resulting alignment was later analyzed with MEGAN6 Community Edition, and a canonical correspondence analysis (CCA) was inferred with the cluster analysis option and a Bray-Curtis ecological distance matrix.

### Rhodopsins

One hundred sixty-eight rhodopsin sequences were extracted from all the metagenomes from assembled contigs longer than 5 Kb. These sequences were pooled with 100 more rhodopsins of fungal, archaeal, viral, and bacterial origin obtained from databases. Sequences were aligned with MUSCLE [107] and a maximum-likelihood tree was constructed with MEGA 7.0 (Jones-Taylor-Thornton model, gamma distribution with five discrete categories, and 100 bootstraps, positions with less than 80% site coverage were eliminated). Blue versus green light absorption was determined as described previously [108]. To compare the abundance of microbial rhodopsins with depth, we initially created a database containing our metagenomic rhodopsin sequences and approximately 7,900 rhodopsin genes obtained from the MicRhoDE database (http://micrhode.sb-roscoff.fr). Metagenomic reads (in subsets of 20 million sequences) were recruited to these rhodopsin sequences using BLASTN (≥ 50 bp alignment, ≥ 99% identity). Rhodopsin sequences that recruited ≥ 1 RPKG were kept for further analyses. In parallel, metagenomic reads were compared to the NR database using DIAMOND (blastx option, top hit, ≥ 50% identity, ≥ 50% alignment length, *E* value < 10^−5^). The abundance of rhodopsin genes in each metagenome was estimated from the number of reads matching rhodopsin sequences in NR, normalized by the number of reads matching *rec*A/*rad*A sequences and by their respective gene length. Reads matching viral or eukaryotic proteins were not taken into account.

### Analysis of glycoside hydrolases

Predicted protein sequences of contigs longer than 5 Kb previously taxonomically classified were compared against the Carbohydrate-Active enZYmes (CAZy) database [64]. Using dbCAN [109], sequences that matched as glycoside hydrolases (GH) with an *E* value < 1e^−8^ were kept for further analyses.

### Functional classification of the assembled proteins

All the proteins encoded within the assembled contigs > 1 Kb were selected, and their putative functionality was inferred against the SEED subsystems [63] and KEGG [110] databases for each metagenome analyzed. Proteins were compared to the SEED database using DIAMOND (blastp option, top hit, ≥ 50% identity, ≥ 50% alignment length, *E* value < 10^−5^). GhostKOALA [111] was used to classify the sequences against the KEGG database.

## Additional files

Additional file 1: **Figure S1.** Method used for sampling. Water was pumped through a hose directly on to the filters instead of using the Niskin bottles rosette. **Figure S2.** Bar plot showing the concentration of inorganic nutrients in both stratified (blue) and mixed (red) samples. **Figure S3.** Simpson Diversity Index versus depth. **Figure S4.** Assembled contigs. A) Size of individual contigs to the left and total assembled size to the right for each phylum. Proteobacteria was divided into its class-level taxonomy. The number of contigs longer than 10 Kb that were taxonomically classified is indicated within brackets. B) Individual contribution of each metagenome to the total assembled size. **Figure S5.** Phylogenetic analysis of Actinobacteria metagenome-assembled genomes (MAGs). A maximum likelihood genome tree was constructed with 100 bootstraps using 31 conserved proteins among the 20 genomes compared. Black circles represent bootstrap values. Between brackets: ANI, average nucleotide identity; COV, percentage of genome sequence shared. In red, those MAGs retrieved in this work. **Figure S6.** Phylogenetic analysis of Alphaproteobacteria MAGs. A maximum likelihood genome tree was constructed with 100 bootstraps using 46 conserved proteins among the 40 genomes compared. Black circles represent bootstrap values. **Figure S7.** Phylogenetic analysis of Bacteroidetes MAGs. A maximum likelihood genome tree was constructed with 100 bootstraps using 21 conserved proteins among the 29 genomes compared. Black circles represent bootstrap values. **Figure S8.** Phylogenetic analysis of Cyanobacteria MAGs. A maximum likelihood genome tree was constructed with 100 bootstraps using 286 conserved proteins among the 45 genomes compared. Black circles represent bootstrap values. **Figure S9.** Phylogenetic analysis of Euryarchaeota MAGs. A maximum likelihood genome tree was constructed with 100 bootstraps using 26 conserved proteins among the 20 genomes compared. Black circles represent bootstrap values. **Figure S10.** Phylogenetic analysis of Gammaproteobacteria MAGs. A maximum likelihood genome tree was constructed with 100 bootstraps using 32 conserved proteins among the 44 genomes compared. Black circles represent bootstrap values. **Figure S11.** Phylogenetic analysis of Verrucomicrobia MAGs. A maximum likelihood genome tree was constructed with 100 bootstraps using 79 conserved proteins among the 25 genomes compared. Black circles represent bootstrap values. **Figure S12.** Phylogenetic analysis of Thaumarchaeota MAGs. A maximum likelihood genome tree was constructed with 100 bootstraps using 129 conserved proteins among the 17 genomes compared. Black circles represent bootstrap values. **Figure S13.** Relative abundance of the reconstructed and reference genomes measured by recruitment (RPKG, reads per kilobase of genome and gigabase of metagenome) from the different depths of the stratified metagenomes. To show the relationships among genomes, a maximum likelihood genome tree was constructed using all the conserved proteins (number in colored square). Each MAG (in blue) has been assigned a name derived from their position in the phylogenomic tree built with the closest known relatives from databases and presented in Additional file 1: Figures S5–S12). Black genomes are from databases (cultures, single amplified genomes, SAGs, or MAGs). **Figure S14.** Phylogenetic analysis of a novel rhodopsin branch of Verrucomicrobia-Planctomycetes superphylum in marine waters. The evolutionary history was inferred by using the maximum likelihood method based on the JTT matrix-based model. A discrete gamma distribution was used to model evolutionary rate differences among sites (five categories). All positions with less than 80% site coverage were eliminated. Sequences in green were isolated from freshwater systems. Colored circles on the right side of sequences indicate the GC content (%) of the contig containing the rhodopsin. Protein sequences were downloaded from NCBI database (www.ncbi.nlm.nih.gov). Tara contigs were downloaded from ENA database (www.ebi.ac.uk/ena). Accession numbers are within brackets. **Figure S15.** Abundance of genes affiliated with membrane transport function based on KEGG modules using principal component analysis (PCA) for each of the individual metagenomics samples. UP, upper photic; DCM, deep chlorophyll maximum; LP, lower photic; MIX, mixed water column (PDF 5881 kb).

Additional file 2: **Table S1.** Relative abundance of 16S rRNA reads. (XLSX 25 kb)

Additional file 3: **Table S2.** Summary statistics of the reconstructed genomes obtained from metagenomes. (XLSX 16 kb)

Additional file 4: **Table S3.** Relative abundance of functional gene categories related to motility and chemotaxis at subsystem level 3 (SEED database). The highest value for each one has been highlighted in red. (XLSX 13 kb)
