# Supplementary material for: Fine metagenomic profile of the Mediterranean stratified and mixed water columns revealed by assembly and recruitment
Source: Microbiome. 2018 Jul 10;6:128. doi: 10.1186/s40168-018-0513-5 (PMC6040077; doi:10.1186/s40168-018-0513-5)
Supplement: Supplementary file 1 — Figure S1. Method used for sampling. Water was pumped through a hose directly on to the filters instead of using the Niskin bottles rosette. Figure S2. Bar plot showing the concentration of inorganic nutrients in both stratified (blue) and mixed (red) samples. Figure S3. Simpson Diversity Index versus depth. Figure S4. Assembled contigs. A) Size of individual contigs to the left and total assembled size to the right for each phylum. Proteobacteria was divided into its class-level taxonomy. The number of contigs longer than 10 Kb that were taxonomically classified is indicated within brackets. B) Individual contribution of each metagenome to the total assembled size. Figure S5. Phylogenetic analysis of Actinobacteria metagenome-assembled genomes (MAGs). A maximum likelihood genome tree was constructed with 100 bootstraps using 31 conserved proteins among the 20 genomes compared. Black circles represent bootstrap values. Between brackets: ANI, average nucleotide identity; COV, percentage of genome sequence shared. In red, those MAGs retrieved in this work. Figure S6. Phylogenetic analysis of Alphaproteobacteria MAGs. A maximum likelihood genome tree was constructed with 100 bootstraps using 46 conserved proteins among the 40 genomes compared. Black circles represent bootstrap values. Figure S7. Phylogenetic analysis of Bacteroidetes MAGs. A maximum likelihood genome tree was constructed with 100 bootstraps using 21 conserved proteins among the 29 genomes compared. Black circles represent bootstrap values. Figure S8. Phylogenetic analysis of Cyanobacteria MAGs. A maximum likelihood genome tree was constructed with 100 bootstraps using 286 conserved proteins among the 45 genomes compared. Black circles represent bootstrap values. Figure S9. Phylogenetic analysis of Euryarchaeota MAGs. A maximum likelihood genome tree was constructed with 100 bootstraps using 26 conserved proteins among the 20 genomes compared. Black circles represent bootstrap values. Figure S10. Phylog [file 40168_2018_513_MOESM1_ESM.pdf]

Fine metagenomic profile of the Mediterranean stratified and mixed water column revealed by assembly and recruitment

Jose M. Haro-Moreno[1,4], Mario López-Pérez[1,4], José R. de la Torre[2], Antonio Picazo[3], Antonio Camacho[3], Francisco Rodriguez-Valera[1*].

[1]Evolutionary Genomics Group, División de Microbiología, Universidad Miguel Hernández, Apartado 18, San Juan 03550, Alicante, Spain. [2]Department of Biology, San Francisco State University, San Francisco, CA 94132, USA. [3]Cavanilles Institute of Biodiversity and Evolutionary Biology, University of Valencia, Burjassot, E-46100 Valencia, Spain. [4]These authors contributed equally to this work.

*Corresponding author: frvalera@umh.es

Evolutionary Genomics Group, División de Microbiología, Universidad Miguel Hernández, Apartado 18, San Juan de Alicante, 03550 Alicante, Spain.

Phone +34-965919313, Fax +34-965 919457

Additional files

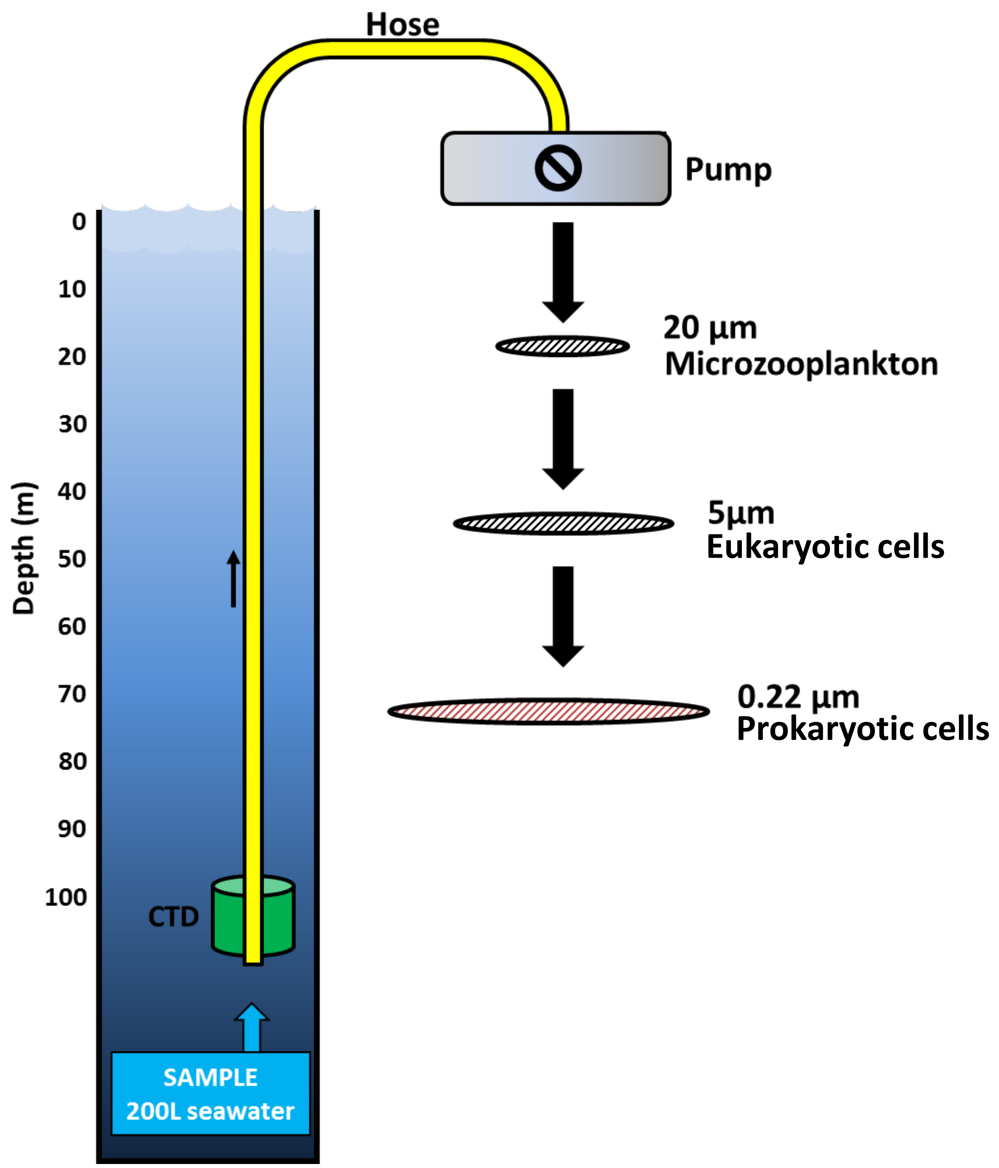

Figure S1. Method used for sampling. Water was pumped through a hose directly on to the filters instead of using the Niskin bottles rossette.

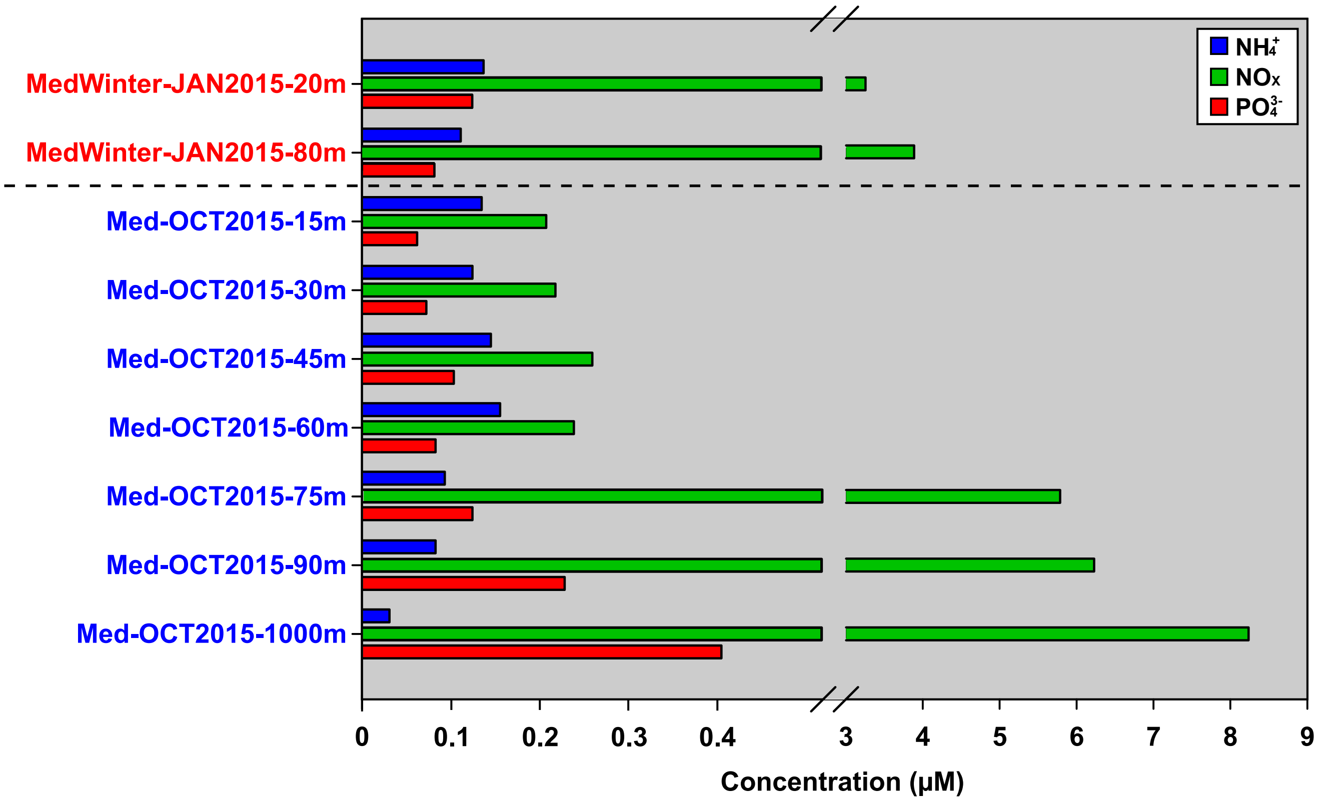

Figure S2. Bar plot showing the concentration of inorganic nutrients in both stratified (blue) and mixed samples (red).

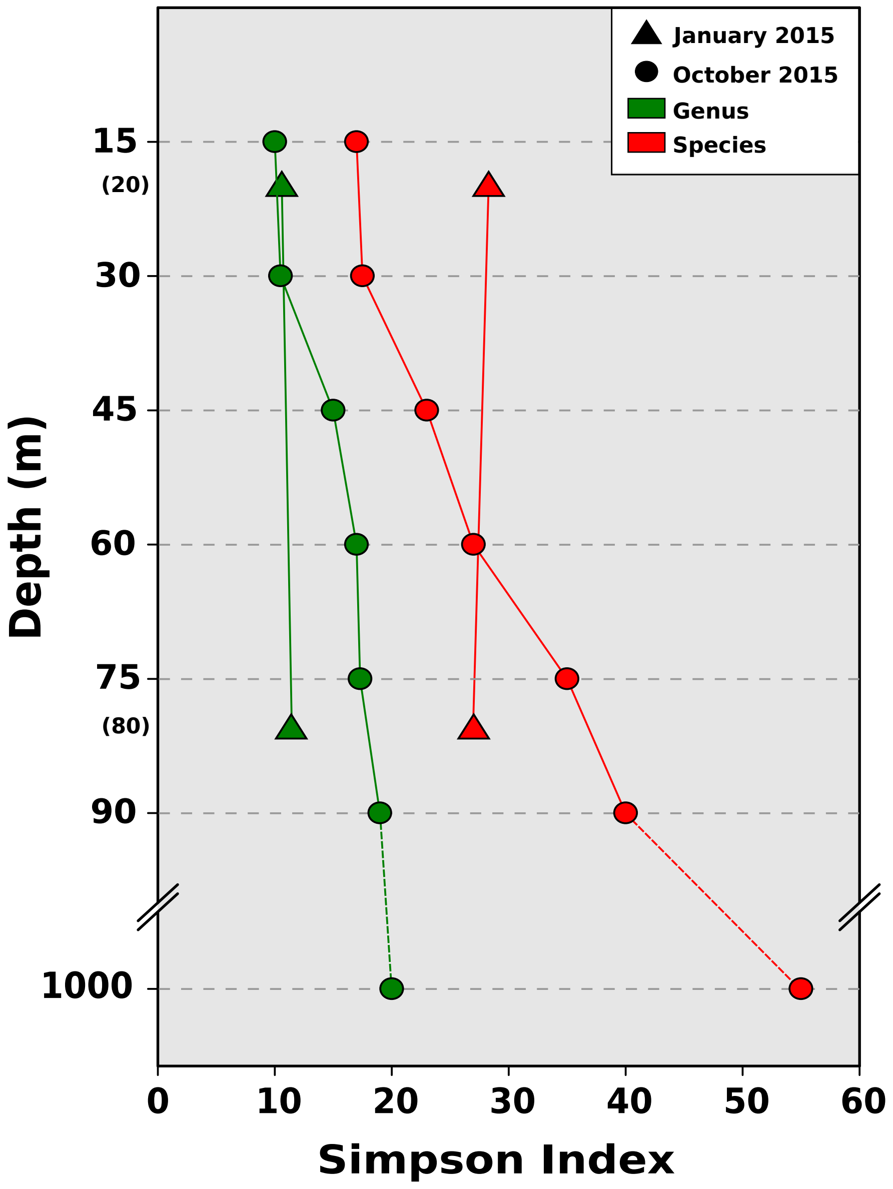

Figure S3. Simpson diversity index versus depth.

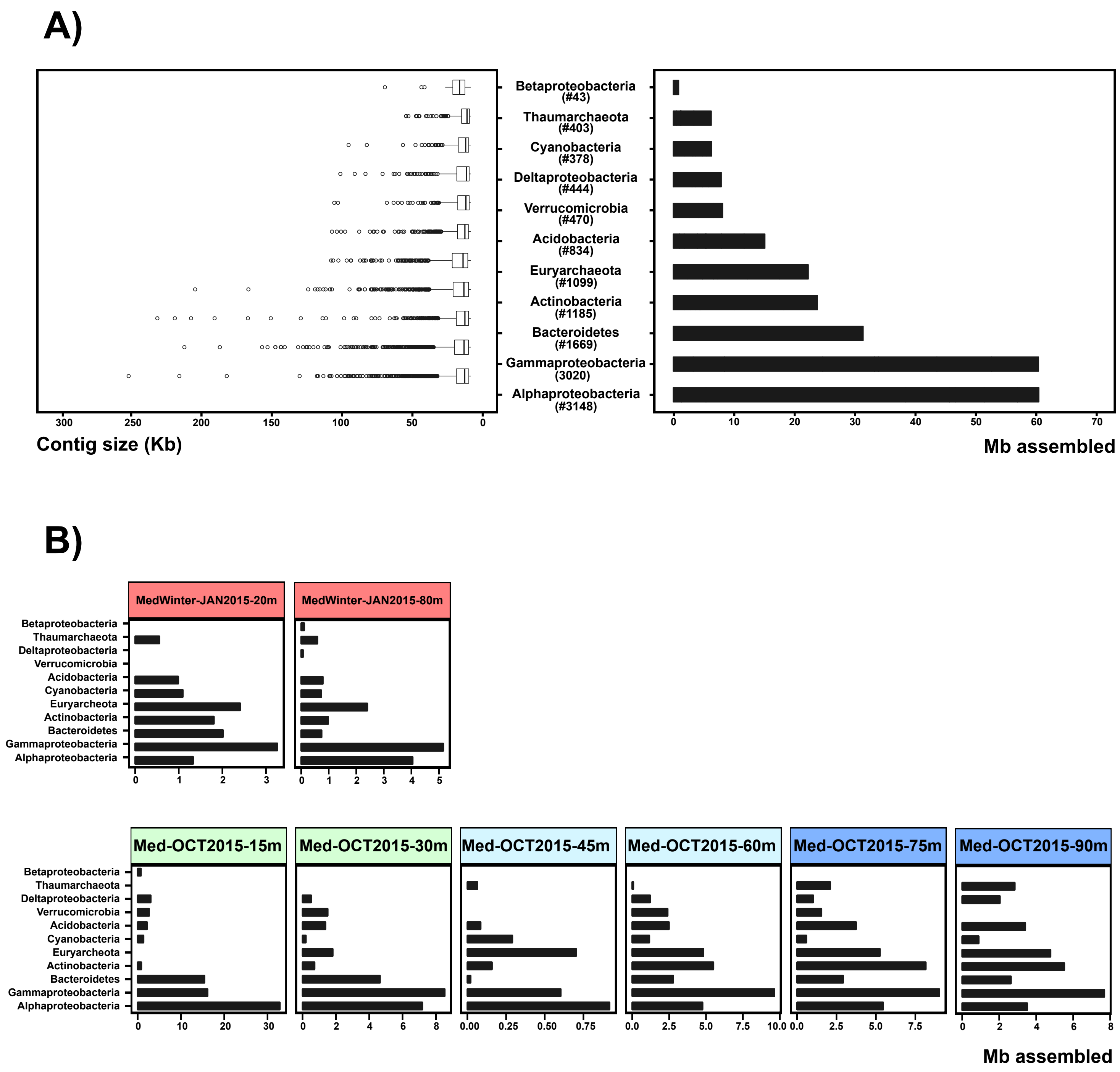

Figure S4. Assembled contigs. A) Size of individual contigs to the left and total assembled size to the right for each phylum. Proteobacteria was divided into its class-level taxonomy. The number of contigs longer than 10 Kb that were taxonomically classified are indicated within brackets. B) Individual contribution of each metagenome to the total assembled size.

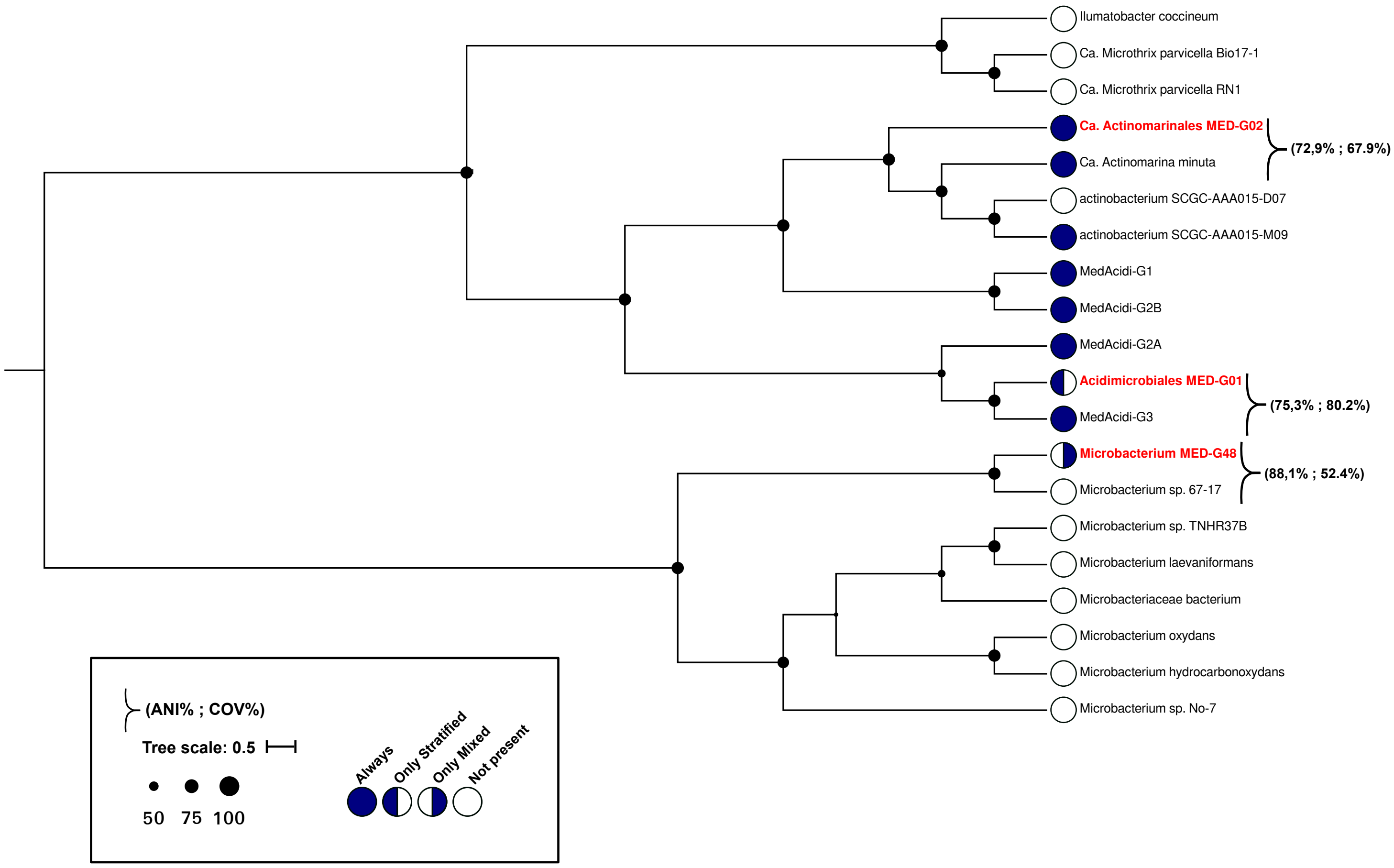

Figure S5. Phylogenetic analysis of Actinobacteria metagenome assembled genomes (MAGs). A maximum likelihood genome tree was constructed with 100 bootstraps using 31 conserved proteins among the 20 genomes compared. Black circles represent bootstrap values. Between brackets: ANI, average nucleotide identity; COV, percentage of genome sequence shared. In red, those MAGs retrieved in this work.

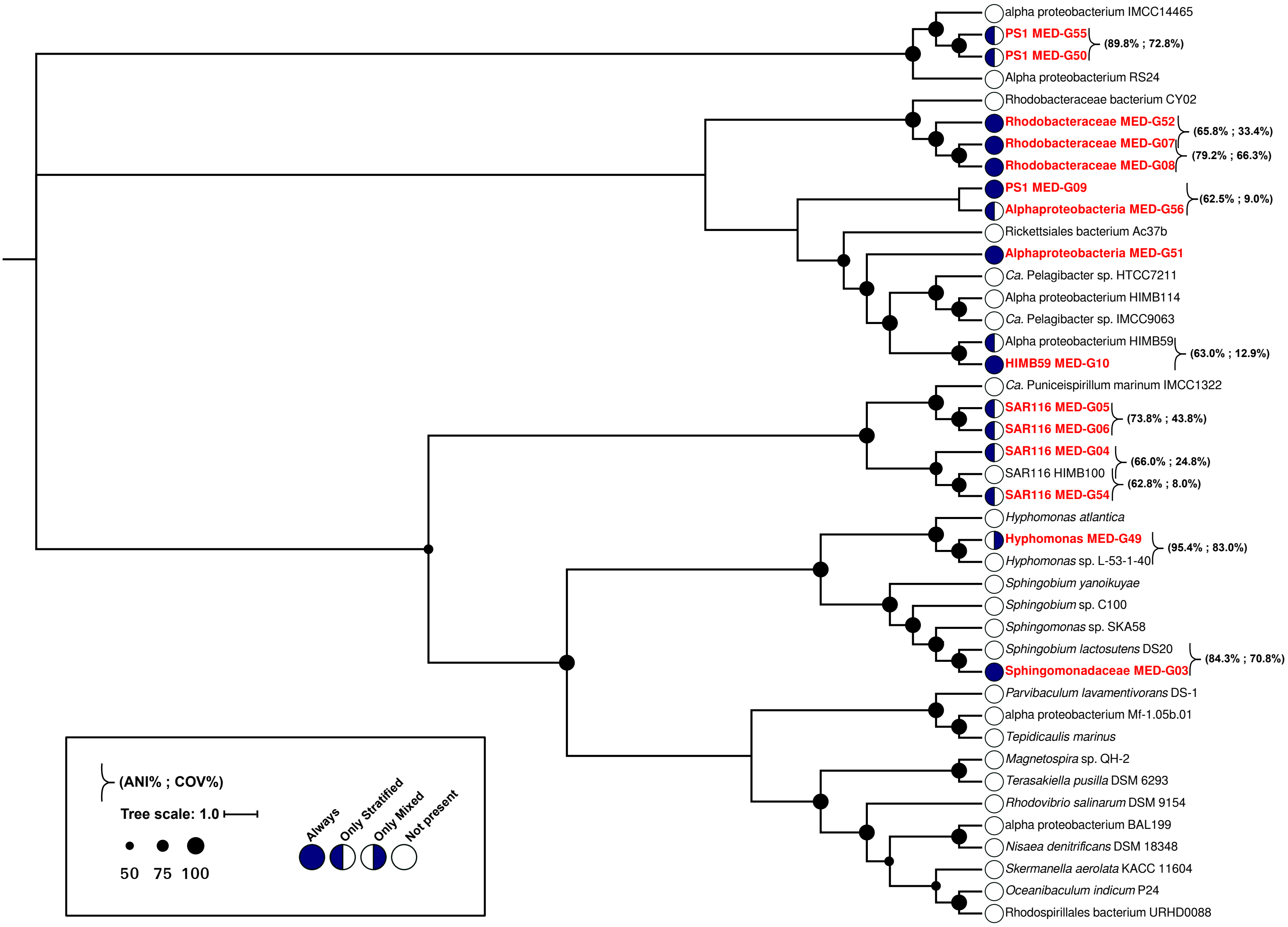

Figure S6. Phylogenetic analysis of Alphaproteobacteria MAGs. A maximum likelihood genome tree was constructed with 100 bootstraps using 46 conserved proteins among the 40 genomes compared. Black circles represent bootstrap values. In red, those MAGs retrieved in this work.

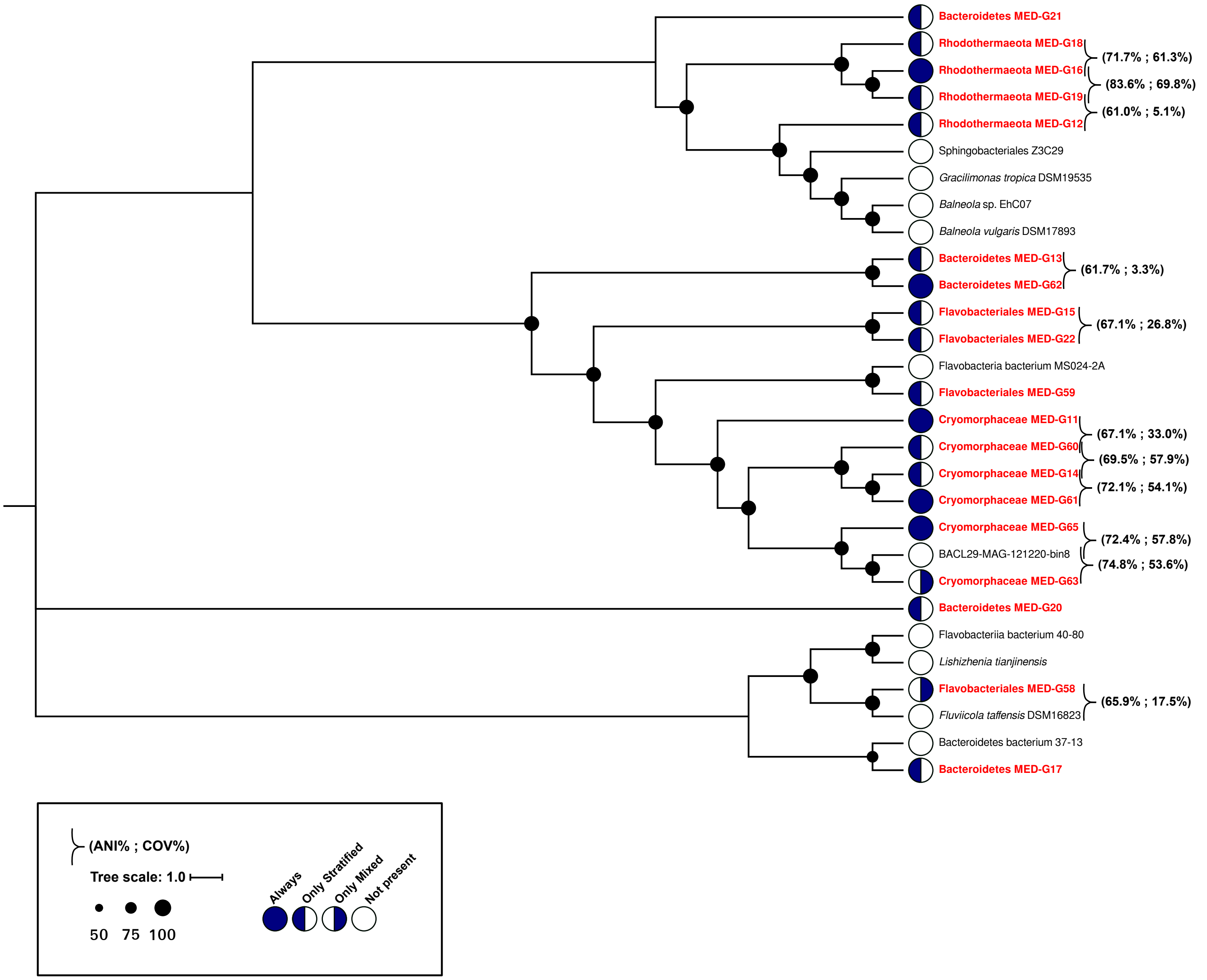

Figure S7. Phylogenetic analysis of Bacteroidetes MAGs. A maximum likelihood genome tree was constructed with 100 bootstraps using 21 conserved proteins among the 29 genomes compared. Black circles represent bootstrap values. In red, those MAGs retrieved in this work.

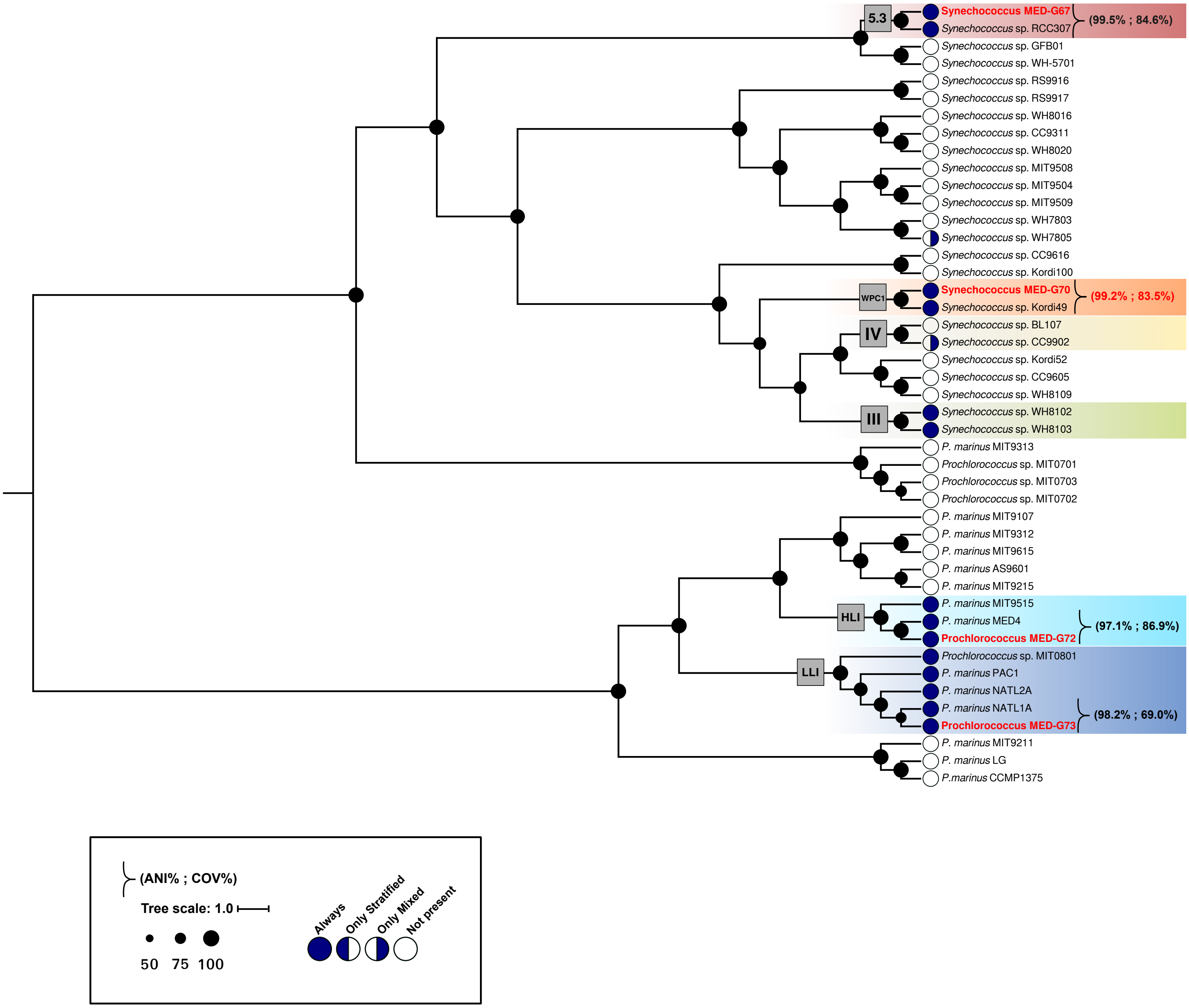

Figure S8. Phylogenetic analysis of Cyanobacteria MAGs. A maximum likelihood genome tree was constructed with 100 bootstraps using 286 conserved proteins among the 45 genomes compared. Black circles represent bootstrap values. In red, those MAGs retrieved in this work.

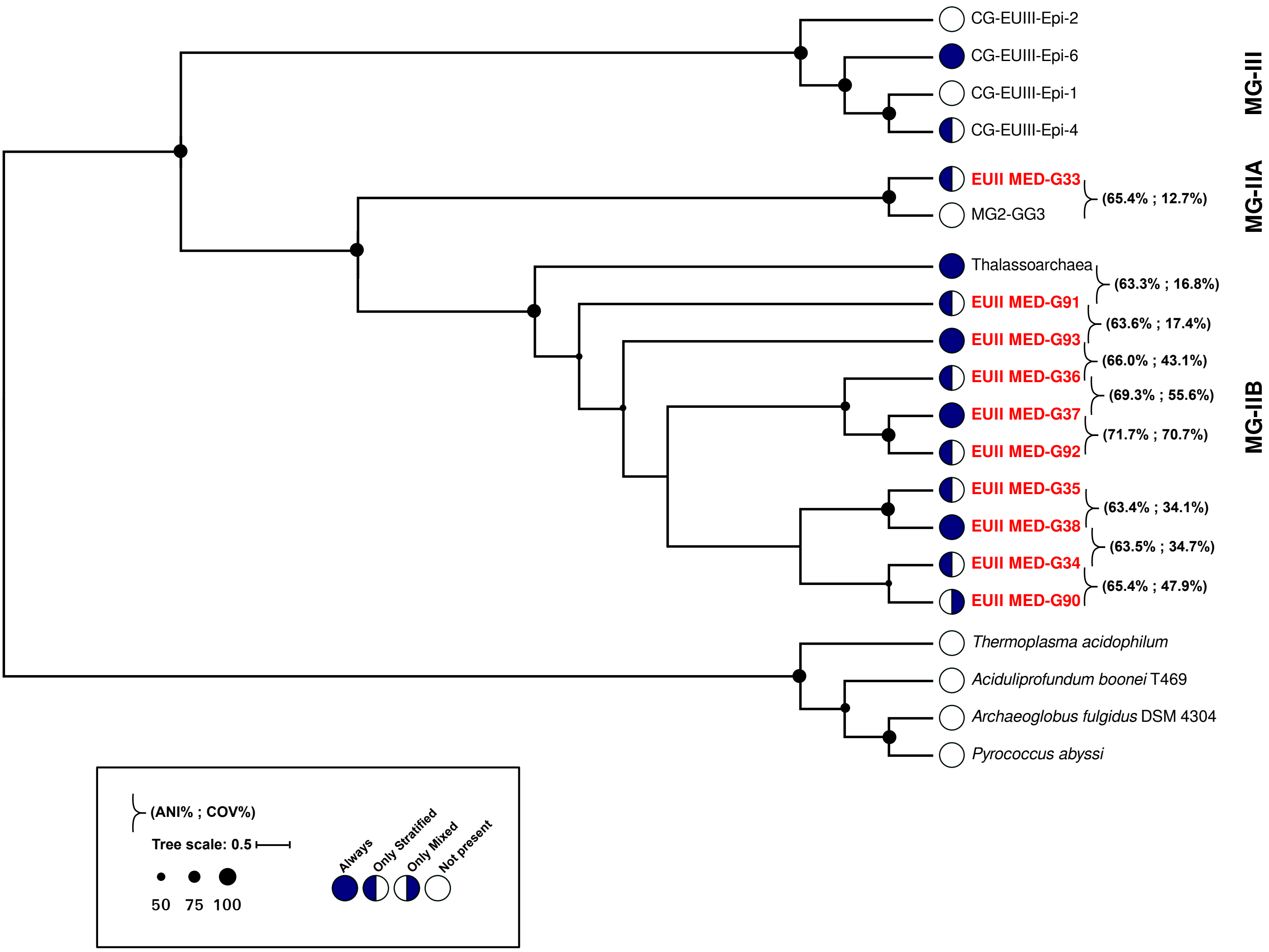

Figure S9. Phylogenetic analysis of Euryarchaeota MAGs. A maximum likelihood genome tree was constructed with 100 bootstraps using 26 conserved proteins among the 20 genomes compared. Black circles represent bootstrap values. In red, those MAGs retrieved in this work.

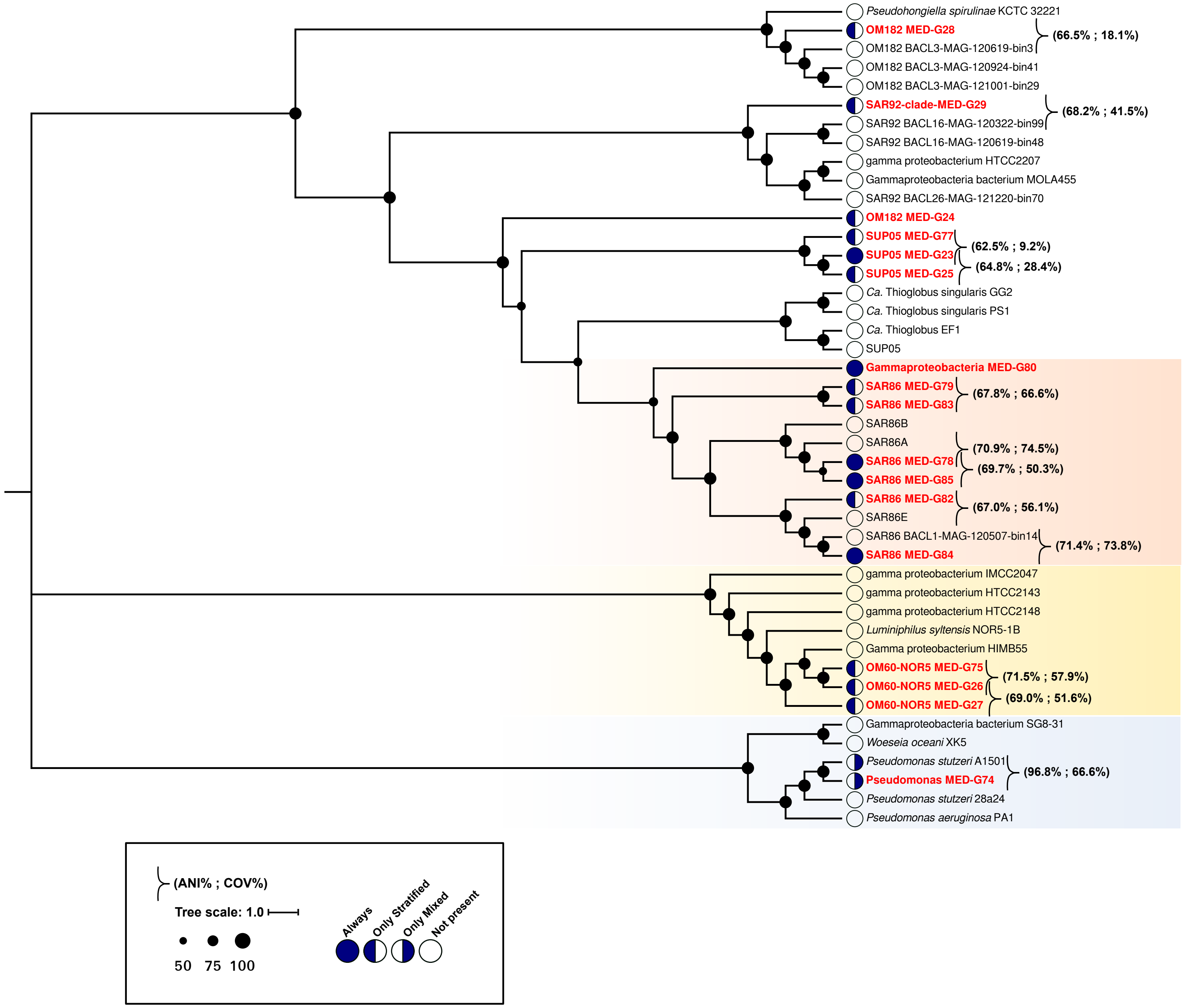

Figure S10. Phylogenetic analysis of Gammaproteobacteria MAGs. A maximum likelihood genome tree was constructed with 100 bootstraps using 32 conserved proteins among the 44 genomes compared. Black circles represent bootstrap values. In red, those MAGs retrieved in this work.

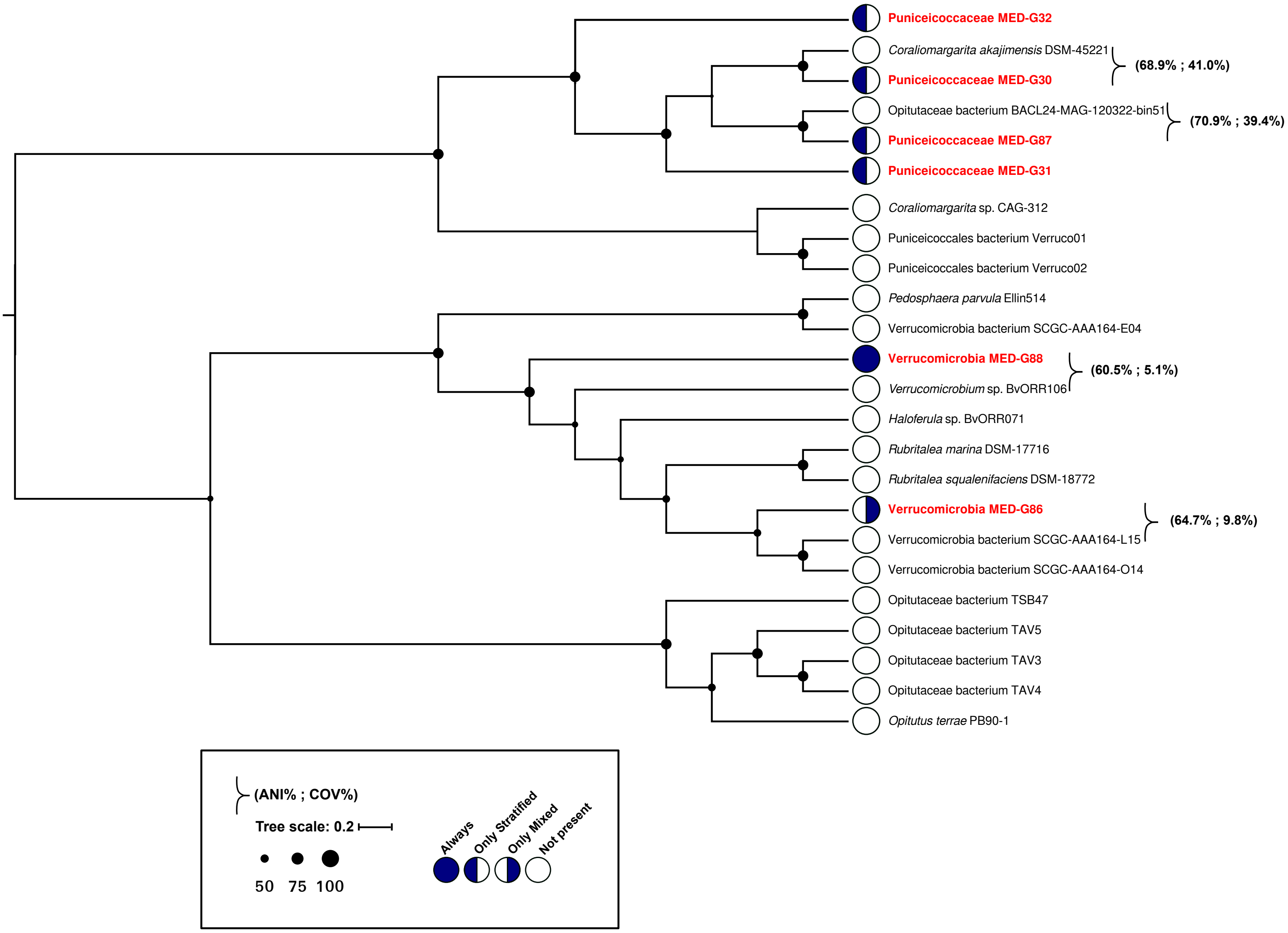

Figure S11. Phylogenetic analysis of Verrucomicrobia MAGs. A maximum likelihood genome tree was constructed with 100 bootstraps using 79 conserved proteins among the 25 genomes compared. Black circles represent bootstrap values. In red, those MAGs retrieved in this work.

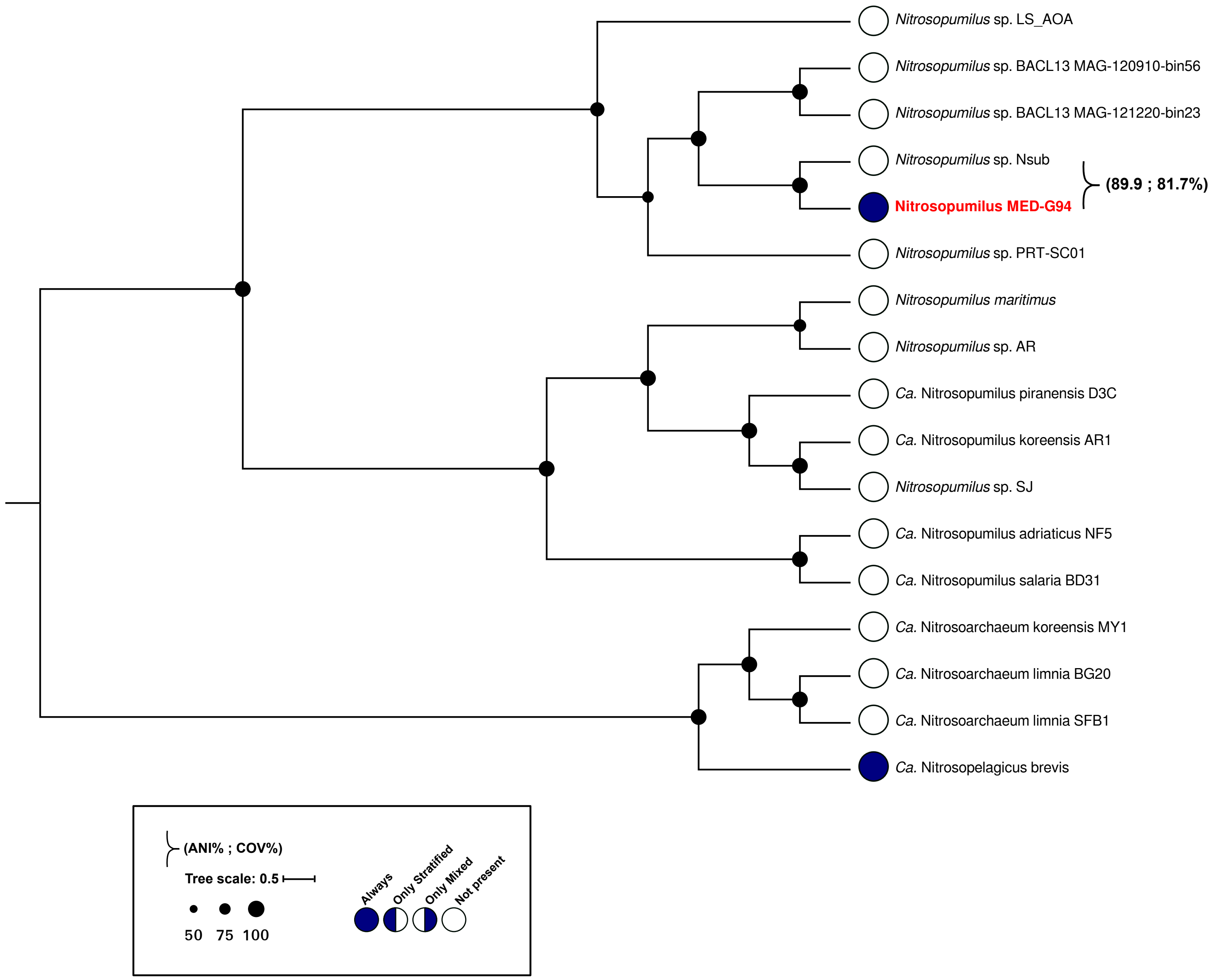

Figure S12. Phylogenetic analysis of Thaumarchaeota MAGs. A maximum likelihood genome tree was constructed with 100 bootstraps using 129 conserved proteins among the 17 genomes compared. Black circles represent bootstrap values. In red, those MAGs retrieved in this work.

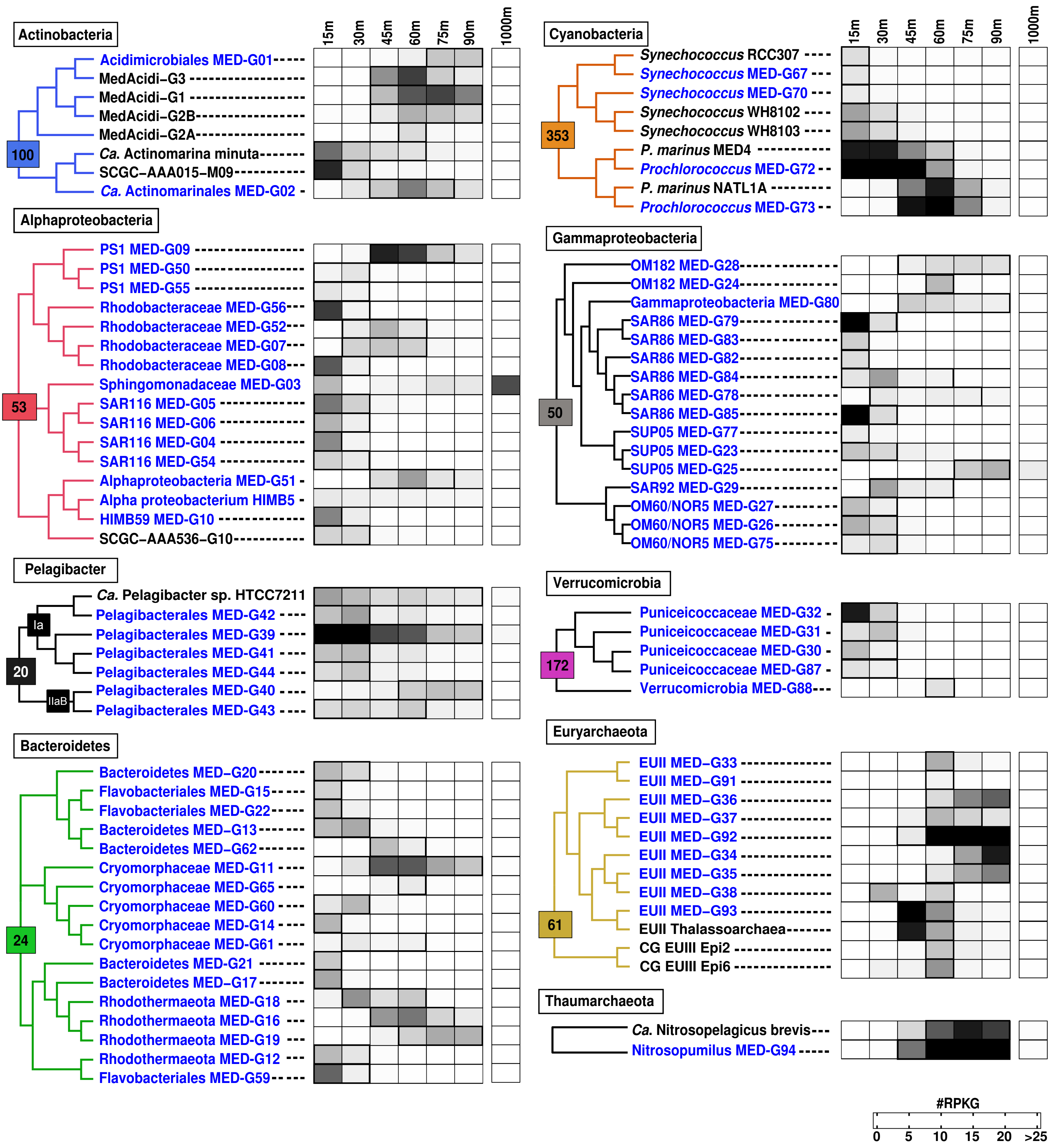

Figure S13. Relative abundance of the reconstructed and reference genomes measured by recruitment (RPKG, Reads per Kilobase of genome and Gigabase of metagenome) from the different depths of the stratified metagenomes. To show the relationships among genomes, a maximum likelihood genome tree was constructed using all the conserved proteins (number in colored square). Each MAG (in blue) has been assigned a name derived from their position in the phylogenomic tree built with the closest known relatives from databases and presented in Figures S5 to S11). Black genomes are from databases (cultures, single amplified genomes, SAGs or MAGs).

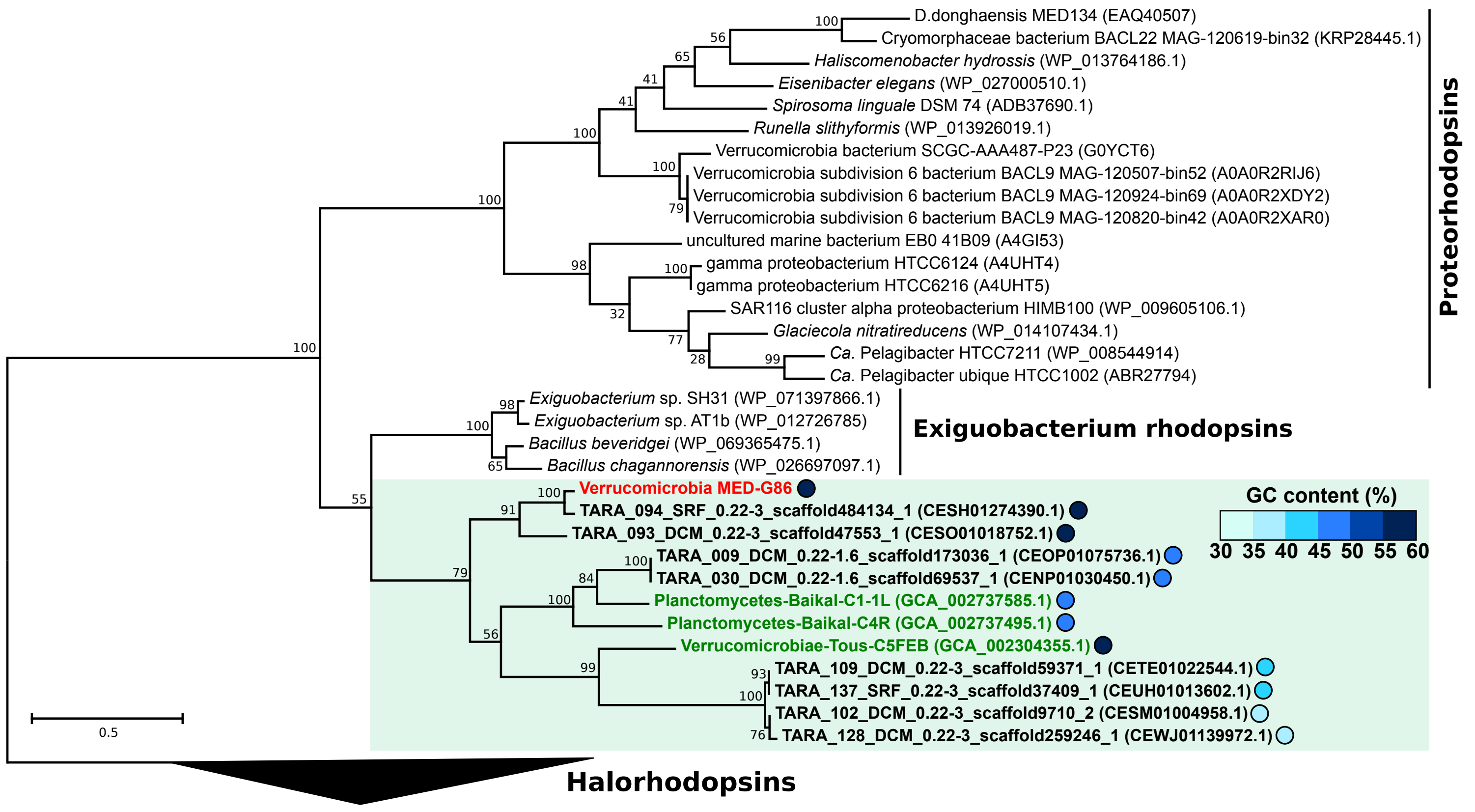

Figure S14. Phylogenetic analysis of a novel rhodopsin branch of Verrucomicrobia-Planctomycetes superphylum in marine waters. The evolutionary history was inferred by using the Maximum Likelihood method based on the JTT matrix-based model. A discrete Gamma distribution was used to model evolutionary rate differences among sites (5 categories). All positions with less than 80% site coverage were eliminated. Sequences in green were isolated from freshwater systems. Colored circles on the right side of sequences indicate the GC content (%) of the contig containing the rhodopsin. Protein sequences were downloaded from NCBI database (www.ncbi.nlm.nih.gov). Tara contigs were downloaded from ENA database (www.ebi.ac.uk/ena). Accession numbers are within brackets.

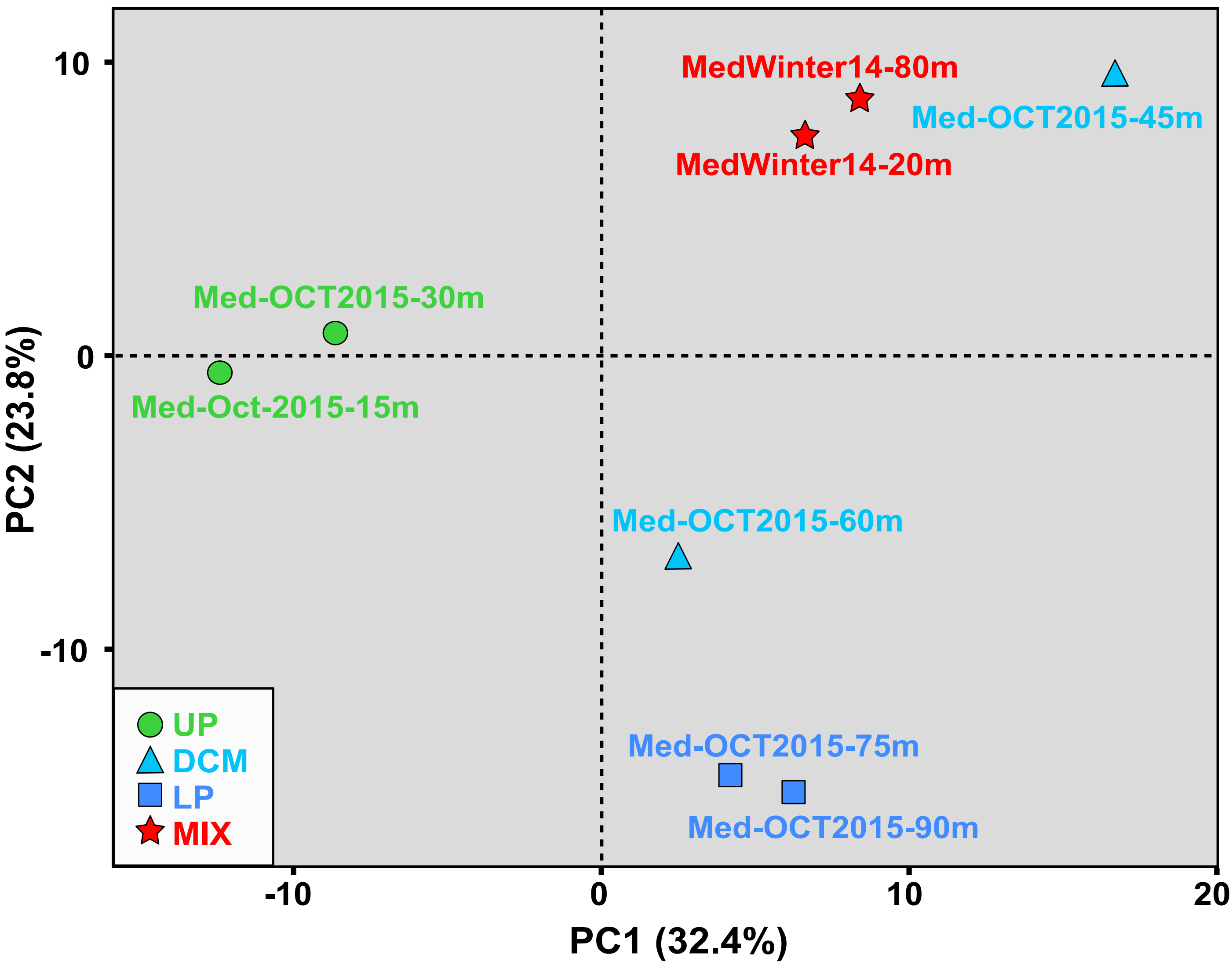

Figure S15. Abundance of genes affiliated to membrane transport function based on KEGG modules using Principal Component Analysis (PCA) for each of the individual metagenomic samples. UP: upper photic, DCM: deep chlorophyll maximum, LP: lower photic and MIX: mixed water column.
